# Modest increase of *KIF11* expression exposes fragilities in the mitotic spindle, causing chromosomal instability

**DOI:** 10.1242/jcs.260031

**Published:** 2022-08-30

**Authors:** Katie L. Dale, Jonathan W. Armond, Robert E. Hynds, Elina Vladimirou

**Affiliations:** ^1^ Cancer Research UK Lung Cancer Centre of Excellence, UCL Cancer Institute, University College London, London, WC1E 6BT, UK; ^2^ Mitotic Dynamics and Chromosomal Instability Laboratory, UCL Cancer Institute, University College London, London, WC1E 6BT, UK; ^3^ Epithelial Cell Biology in ENT Research Group, UCL Great Ormond Street Institute of Child Health, University College London, London, WC1N 1EH, UK

**Keywords:** Chromosomal instability, Aneuploidy, Micronuclei, KIF11, Mitosis, CRISPR activation, Confocal imaging, Machine learning, Deep learning

## Abstract

Chromosomal instability (CIN), the process of increased chromosomal alterations, compromises genomic integrity and has profound consequences on human health. Yet, our understanding of the molecular and mechanistic basis of CIN initiation remains limited. We developed a high-throughput, single-cell, image-based pipeline employing deep-learning and spot-counting models to detect CIN by automatically counting chromosomes and micronuclei. To identify CIN-initiating conditions, we used CRISPR activation in human diploid cells to upregulate, at physiologically relevant levels, 14 genes that are functionally important in cancer. We found that upregulation of *CCND1*, *FOXA1* and *NEK2* resulted in pronounced changes in chromosome counts, and *KIF11* upregulation resulted in micronuclei formation. We identified KIF11-dependent fragilities within the mitotic spindle; increased levels of KIF11 caused centrosome fragmentation, higher microtubule stability, lagging chromosomes or mitotic catastrophe. Our findings demonstrate that even modest changes in the average expression of single genes in a karyotypically stable background are sufficient for initiating CIN by exposing fragilities of the mitotic spindle, which can lead to a genomically diverse cell population.

## Teaser

**Highlighted Article:** Using a high-throughput, single-cell, image-based pipeline to detect chromosomal instability, we found that modest *KIF11* upregulation compromised mitotic spindle integrity and induced aneuploidy via micronuclei formation.

## INTRODUCTION

Chromosomal instability (CIN) is the dynamic process of gains or losses of chromosomes or parts of chromosomes at an elevated rate. CIN results in aneuploid cells, which contain an abnormal complement of chromosomes. CIN is an ongoing process and is distinct from aneuploidy (a static state) and mis-segregation (a one-off, stochastic event) (Gordon et al., 2012). In cancer, CIN promotes intratumour heterogeneity and evolution by enabling the rapid exploration of genotypes, and correlates with drug resistance and poor patient survival (Sansregret et al., 2018). In ageing, age-associated mitotic dysfunction underlies low-level CIN, which gives rise to aneuploid senescent cells. In conjunction with a less effective immune response, this can induce a pro-inflammatory state associated with age-related diseases, including a susceptibility to cancer initiation (Barroso-Vilares and Logarinho, 2019). Therefore, CIN compromises genomic integrity with profound consequences to human health.

Numerical CIN has been attributed to defects in the mitotic machinery, including erroneous kinetochore–microtubule attachments, supernumerary centrosomes, an impaired spindle assembly checkpoint and impaired sister chromatid cohesion (Gordon et al., 2012; Thompson et al., 2010). Cell-biological studies have identified perturbations that compromise the mitotic machinery, giving rise to chromosome mis-segregation or aneuploidy (Ricke et al., 2011; Shrestha et al., 2021; Sotillo et al., 2007; Zhang et al., 2008). Although many studies use supraphysiological overexpression, modest elevation of gene expression has not been widely investigated. Indeed, RNA sequencing studies have revealed moderate, yet functionally important, upregulation of gene expression in cancer (Biswas et al., 2019; Teixeira et al., 2019). Moreover, *de novo* CIN can be masked in studies that use CIN-positive cell lines or cancer tissues with diverse and fluid karyotypes. Similarly, CIN-initiating events can occur with low probability, rendering them hard or impossible to detect in bulk assays. Therefore, our understanding of genes that are capable of triggering CIN with only moderate upregulation remains limited.

We developed a high-throughput, single-cell, image-based pipeline to detect CIN using CRISPR activation to model moderate upregulation of single genes in retinal pigment epithelial-1 (RPE1) cells, a non-transformed, diploid, epithelial cell line. Using CRISPR, we activated 14 genes that were identified in early non-small-cell lung cancer (Jamal-Hanjani et al., 2017; Teixeira et al., 2019) or are overexpressed in other cancers (Daigo et al., 2018; Zhang et al., 2010). We found that the upregulation of *CCND1*, *FOXA1*, *NEK2*, *MAD2L1*, *PIK3CA*, *KIF18A* and *KIF11* induced CIN, with upregulation of *CCND1*, *FOXA1* and *NEK2* having the biggest effect and *KIF11* upregulation showing a pronounced increase in the number of micronuclei. We analysed the *KIF11* upregulation-induced CIN and found that an average fourfold increase in gene expression caused centrosome fragmentation and gave rise to CIN owing to a force imbalance, which could be rescued by *HSET* (also known as *KIFC1*) activation. Our data show that even modest single-gene expression changes on average are a sufficient condition for initiating CIN.

## RESULTS

### Pipeline for automated extraction of centromere and micronuclei counts from single-cell high-throughput imaging

**Fig. 1.**
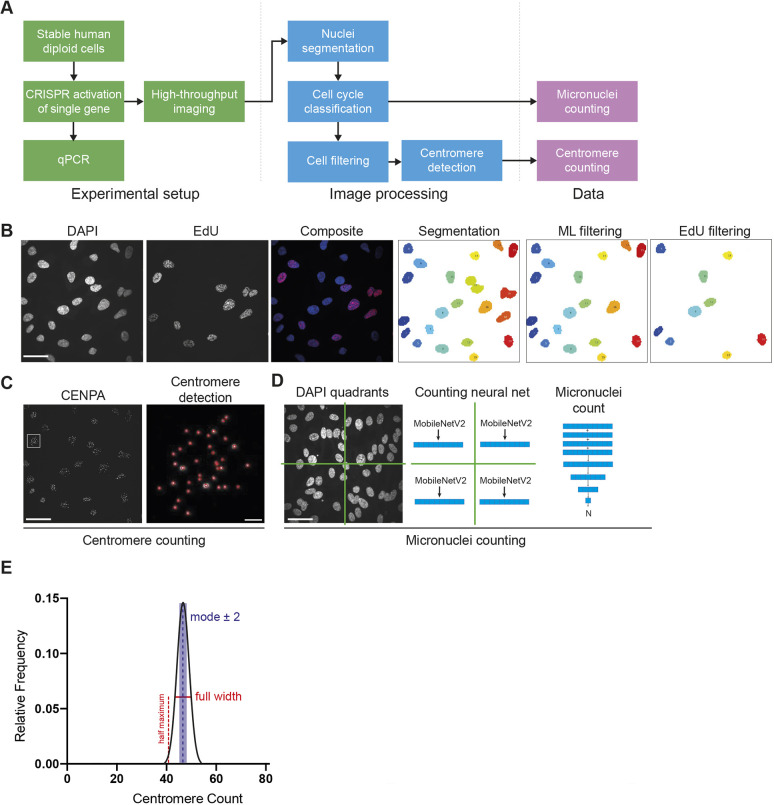
**Development of a high-throughput single-cell imaging pipeline to detect CIN.** (A) Flow chart detailing the experimental and image-processing stages of the pipeline for automated counting of micronuclei and quantification of numbers of centromeres from single nuclei. (B) Example of an imaged field of cells and the stages of imaging processing. Left to right: nuclei of cells indicated by DAPI staining; cells in S phase identified by EdU staining; a composite image showing a cell population at different cell cycle stages; segmentation of nuclei and automatic assignment of IDs as shown in different colours; machine learning (ML)-based filtering of nuclei to exclude cells that touch, cells that are found on the edge of the imaging field and cell debris; and EdU thresholding to exclude S phase cells. (C) For centromere counting, centromeres were stained with an antibody against CENP-A, an H3 variant found in all centromeres. Magnification of a single nucleus (white square) is shown on the right. Using the CENP-A signal, centromeres are automatically detected and shown as the red circles surrounding the CENP-A signal in greyscale. (D) For micronuclei counting, the DAPI images are divided into quadrants to ensure sufficient image resolution for the algorithm requirements. Using a counting neural network, the micronuclei from each quadrant are counted, and the total number of micronuclei of the original field is calculated by summing up the micronuclei from each quadrant. (E) Two measurements from the frequency plots are used to quantify the occurrence and degree of CIN from centromere counts: FWHM (in red) and the proportion of cells deviating from the modal number of centromeres (±2 centromeres, in blue). Scale bars: 30 μm (B); 30 μm (C, left); 2 μm (C, right); 30 μm (D).

To detect perturbations that could result in CIN following the activation of the 14 genes we targeted by CRISPR, our imaging assay for CIN detection involved multiple stages (Fig. 1A): (1) an experimental setup for targeted activation of a single gene and subsequent high-throughput confocal imaging of fixed cells, (2) an automated image-processing step comprising the use of a deep neural network for nuclei segmentation, cell-cycle-phase classification and a centromere-detection algorithm (Fig. 1B), and (3) an analysis step using a deep neural network for micronuclei (MN) counting directly from images and a centromere-counting algorithm (Fig. 1C,D).

Cells were fixed and stained with an antibody against CENP-A (a centromere-specific histone H3 variant), DAPI and the thymidine analog 5-ethynyl-2′-deoxyuridine (EdU). The latter was used to detect and exclude cells in S phase with already replicated centromeres, which would bias the centromere counting, and to only count the number of centromeres in cells that were in G1. Subsequently, we used high-throughput confocal microscopy to acquire thousands of three-dimensional (3D) multichannel images of cells per condition.

To quantify CIN and MN numbers, we used our automated image-analysis pipeline (see Materials and Methods). From the frequency plots of the centromere counts, we used two measurements: the full width at half maximum (FWHM) (in red, Fig. 1E) and the proportion of cells deviating from the mode by ±2 centromeres (in blue, Fig. 1E). The FWHM measures the width of the distribution by joining two points on the curve at half the maximum height. The modal deviation is an extension of a previously used method to assess aneuploidy with chromosome-specific fluorescence *in situ* hybridisation (FISH) probes (Bakhoum et al., 2009).

### Validation of pipeline using pharmacologically induced CIN

**Fig. 2.**
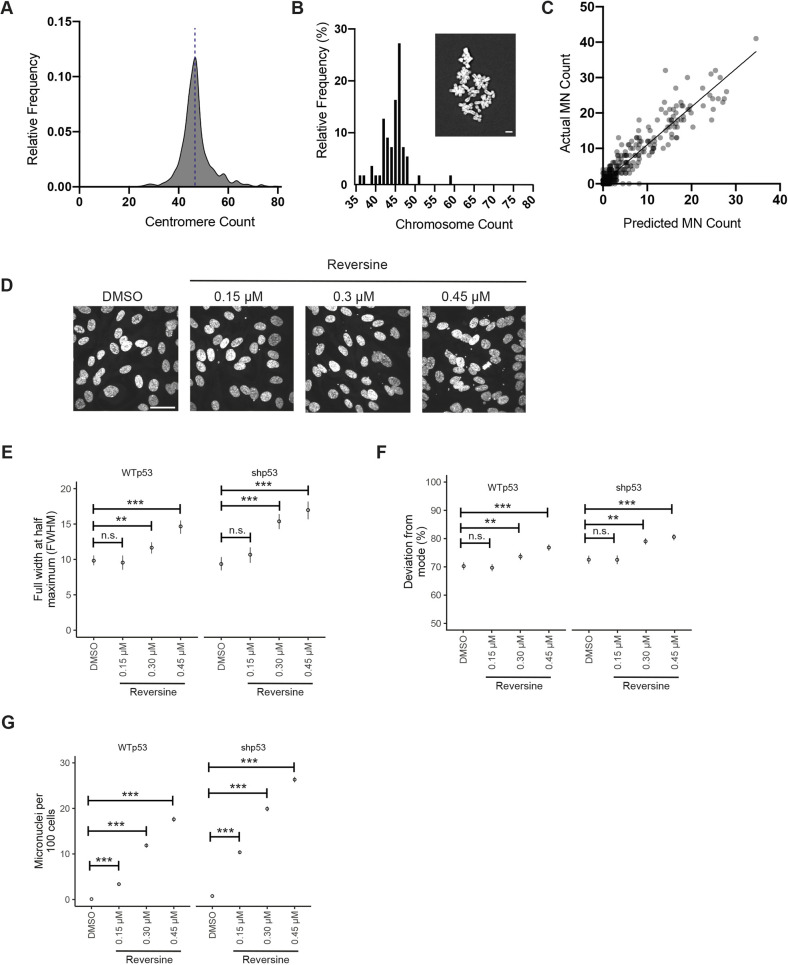
**Validation of the CIN detection pipeline.** (A) Frequency plot of the automated centromere counting of untreated RPE1 cells. The dotted blue line indicates the diploid count of 46 chromosomes. (B) Histogram showing chromosome counts from metaphase spreads of RPE1 cells with an example image (inset), *n*=28. (C) Predicted micronuclei counts plotted against the actual micronuclei counts from a set of manually labelled images to evaluate the accuracy of the algorithm (Pearson's correlation coefficient r_p_=0.88, *P*<0.0005, *n*=475, four experiments). (D) Images of DAPI-stained cells treated with DMSO and varying doses of reversine showing increasing numbers of micronuclei with increasing concentrations of reversine. (E) FWHM of centromere-count frequency plots of DMSO and reversine-treated RPE1 (wild type or WT) and RPE1-p53-shRNA cells (permutation test). (F) Percentage of centromere counts that deviate from the mode from DMSO and reversine-treated RPE1 (WT) and RPE1-p53-shRNA cells (permutation test). (G) Number of micronuclei per 100 cells in DMSO and reversine-treated RPE1 (WT) and RPE1-p53-shRNA cells (two-tailed unpaired *t*-test). Data show the mean±s.e.m. *n*=6033, 6131, 5880, 5536 over four replicates for DMSO, 0.15 μM, 0.30 μM and 0.45 μM reversine, respectively, for E–G. n.s., not significant; **P<0.005; ***P<0.0005. Scale bars: 5 μm (B); 30 μm (D).

Using the experimental protocol outlined above, we first checked the accuracy of the automated centromere counting using untreated RPE1 cells, which are near diploid. We found a modal centromere count of 45 with some variation around this number (Fig. 2A), which reflects both the variability in chromosome counts as well as noise introduced by the multiple steps of the analysis pipeline, which we expect to be unbiased. We performed metaphase spreads of untreated RPE1 cells and found a variation in chromosome counts around the modal chromosome count of 46 (Fig. 2B), so it is likely that at least a proportion of these deviations from true diploidy reflects genuine variation of chromosome number in the cell line. The discrepancy of a single centromere between the counting algorithm and the metaphase spreads is indicative of our conservative approach towards resolving superimposed centromere spots. To evaluate the accuracy of the micronuclei-counting algorithm, we plotted the predicted micronuclei counts against the manually observed micronuclei counts from a set of images with a mean absolute error of 1.0 (Fig. 2C; Pearson's correlation coefficient r_p_=0.88, *P*<0.0005, two-tailed one-sample *t*-test). In comparison with the accuracy described in the literature for other methods for automatically counting micronuclei, we achieved higher sensitivity compared to Metafer MNScore (Varga et al., 2004), Pathfinder (Decordier et al., 2009) and others (Bahreyni Toossi et al., 2017), with reported sensitivities of 35%, 69% and 82%, respectively. In a study on human peripheral lymphocytes, Cell Profiler was used to automatically detect micronuclei, with a reported overestimation of 33% (Ramadhani and Purnami, 2013). In comparison, our algorithm has high sensitivity (86.9%), low mean absolute error (1.0) and is high throughput.

To determine the sensitivity of our assay to detect CIN, we pharmacologically induced chromosome mis-segregation by treating RPE1 cells with increasing doses of reversine, an inhibitor of the spindle assembly checkpoint (SAC) kinase MPS1, which increases the rate of chromosome mis-segregation in a dose-dependent manner but does not lead to arrest in the subsequent G1 for most cells (Sansregret et al., 2017; Santaguida et al., 2010). To enable more chromosome mis-segregation events to occur, we also performed the reversine titration experiment in RPE1 cells transduced with a lentiviral construct encoding an shRNA, which causes degradation of p53 (encoded by *TP53*) mRNA (Godar et al., 2008). After 24 h of treatment with increasing doses of reversine or DMSO (vehicle), both experiments were fixed, imaged and analysed as described above to automatically quantify the number of centromeres in individual cells and the number of micronuclei (Fig. 2D). For RPE1 cells, treatment with 0.3 μM and 0.45 μM reversine resulted in larger FWHM, which is dose-dependent and is suggestive of aneuploidy, whereas treatment with 0.15 μM reversine did not show detectable changes (Fig. 2E; FWHM of 9.5, 11.7 and 14.7 for 0.15, 0.3 and 0.45 μM reversine, respectively, compared to 9.8 for DMSO; *P*=0.65, *P*=0.003 and *P*<0.0005, respectively; permutation test). In RPE1-p53-shRNA cells, we found the same dose-dependent aneuploidy trend, which was exacerbated by the absence of p53 (Fig. 2E; FWHM of 10.7, 15.4 and 17 for 0.15, 0.3 and 0.45 μM reversine, respectively, compared to 9.4 for DMSO; *P*=0.06, *P*<0.0005 and *P*<0.0005, respectively; permutation test). In both RPE1 and RPE1-p53-shRNA cells treated with increasing doses of reversine, the deviation from the mode increased in a dose-dependent manner in a similar trend as the FWHM (Fig. 2F; for RPE1 cells: 70%, 74% and 77% for 0.15, 0.3 and 0.45 μM reversine, respectively, compared to 70% for DMSO; *P*=0.006, *P*=0.003 and *P*=0.5, respectively. For RPE1-p53-shRNA cells: 72%, 79% and 81% for 0.15, 0.3 and 0.45 μM reversine, respectively, compared to 73% for DMSO; *P*=0.96, *P*=0.0006 and *P*<0.0005, respectively; all permutation tests). Previous studies have shown that whole-chromosome mis-segregation rarely leads to p53 activation (Santaguida and Amon, 2015; Soto et al., 2017); thus, to avoid subjecting the cells to multiple transductions, we carried out all our subsequent experiments in p53-proficient cells. Analysis of the reversine titration experiments using our micronuclei-counting algorithm revealed a reversine-dose-dependent increase in the number of micronuclei (reported as micronuclei per 100 cells) for all three doses of reversine (Fig. 2G; for RPE1 cells: 3.4, 11.9 and 17.6 MN/100 cells for 0.15, 0.3 and 0.45 μM reversine, respectively, compared to 0.1 MN/100 cells for DMSO; *P*<0.0005 for all conditions. For RPE1-p53-shRNA cells: 10.4, 19.9 and 26.3 MN/100 cells for 0.15, 0.3 and 0.45 μM reversine, respectively, compared to 0.8 MN/100 cells for DMSO; *P*<0.0005 for all conditions; two-tailed unpaired *t*-tests). These validation experiments confirmed that the two-stage pipeline we have developed for both automated centromere and micronuclei counting is able to robustly detect CIN and aneuploidy using single-cell high-throughput imaging.

### Moderate upregulation of single genes is sufficient to initiate CIN in human diploid cells

**Fig. 3.**
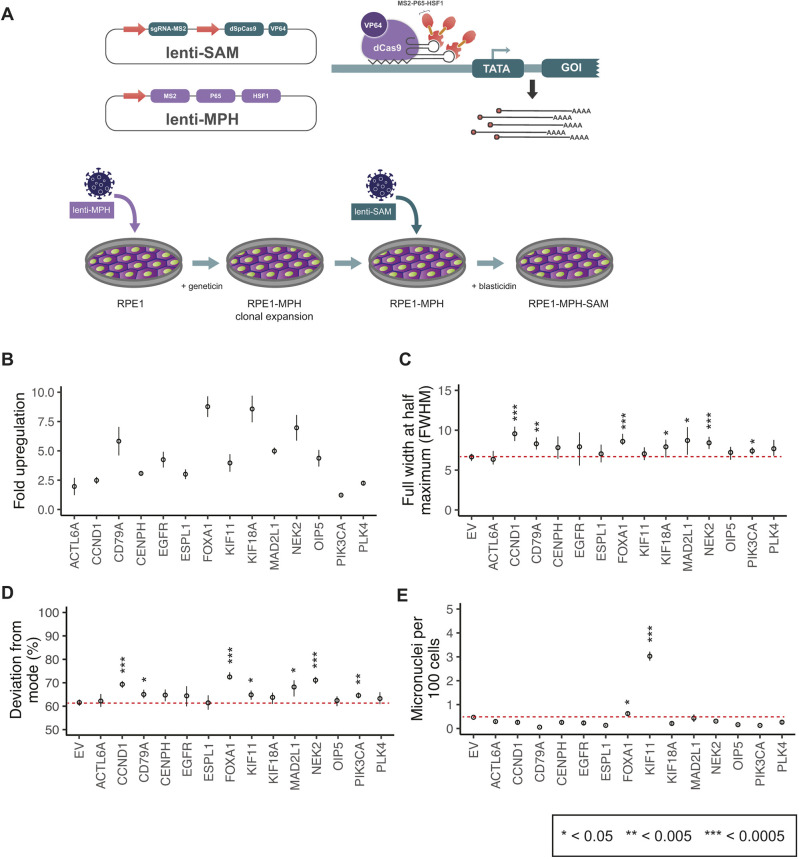
**CIN detection after single-gene upregulation with CRISPR activation.** (A) Schematic of the Synergistic Activation Mediators (SAM) CRISPR activation system used to upregulate single genes in RPE1 cells, one gene at a time, with an outline of the experimental protocol used to incorporate the components required for the activation complex. (B) Fold upregulation measured by RT-qPCR after CRISPR activation of a single gene in each cell population. (C) FWHM of centromere-count frequency plots from cells with a CRISPR activated gene (permutation test). (D) Percentage of centromere counts that deviate from the mode from cells with a CRISPR-activated gene (permutation test). (E) Number of micronuclei per 100 cells upon CRISPR activation of single genes (two-tailed unpaired *t*-test). Data show the mean±s.e.m. Dotted red lines in C–E indicate the respective values measured for treatment with the empty vector (EV). Cell counts for C–E are shown in Table 1. *P<0.05; **P<0.005; ***P<0.0005.

We next investigated whether the moderate upregulation of single genes is sufficient to initiate CIN. We incorporated the Synergistic Activation Mediators (SAM) System, a CRISPR activation (CRISPRa) system (Konermann et al., 2015), into RPE1 cells. This system mediates transcriptional activation by recruiting multiple sets of transcription factors and chromatin remodelling complexes at endogenous genomic loci, which work synergistically to upregulate a gene of interest (Fig. 3A) (Konermann et al., 2015).

**Table 1.**
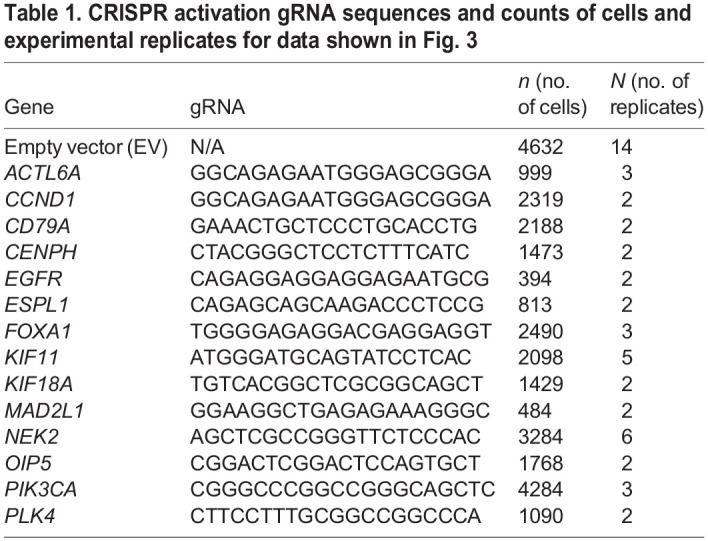
CRISPR activation gRNA sequences and counts of cells and experimental replicates for data shown in Fig. 3

Using the guide RNA selection tool CRISPOR (Concordet and Haeussler, 2018), we designed 5–8 single guide RNAs (sgRNAs) per gene, targeting 14 genes that are either amplified during the early stages of cancer or are upregulated in cancer. Using high-throughput cloning and a lentiviral expression system, we established expression vectors and lentiviral particles for sgRNA-dCas9-VP64 for each guide of each gene. We generated an RPE1 clonal cell line expressing the MS2–p65–HSF1 (hereafter MPH) protein, which would form the RPE1-MPH starting cell line for all the CRISPRa experiments. To assess the ability of the RPE1-MPH cell line to allow CIN propagation, we pharmacologically induced CIN using a combination of CENP-E and MPS1 inhibitors, shown to specifically induce numerical CIN (Soto et al., 2017). Both combination doses that were tested caused significant increases in FWHM, deviations from the modal number of centromere counts and numbers of micronuclei (Fig. S1A–C). Transductions in RPE1-MPH cells resulted in robust gene upregulation, whereas simultaneous transduction of the RPE1 cell line with MPH and SAM did not (Fig. S1D). For each target gene, we identified the single guide that consistently resulted in gene upregulation (Fig. S1E). From 5 to 7 days post transduction, we assessed gene upregulation by quantitative reverse transcription PCR (RT-qPCR) and found successful consistent upregulation of all 14 genes, which was guide dependent (1.2- to 8.8-fold upregulation; Fig. 3B). We also confirmed that CRISPRa resulted in sustained increased levels of mRNA and protein during the time course of 4, 7 and 14 days (Fig. S1F,G).

We then fixed and imaged cells using the protocol outlined in Fig. 1 and analysed them using the automated pipeline. We found that upregulation of *CCND1*, *CD79A*, *FOXA1*, *KIF18A*, *MAD2L1*, *NEK2* and *PIK3CA* increased FWHM, indicative of wider distribution of centromere counts around diploidy compared to that of cells transduced with an empty vector (EV^CRISPRa^; Fig. 3C). Upregulation of *CCND1*, *CD79A*, *FOXA1*, *KIF11*, *MAD2L1*, *NEK2* and *PIK3CA* resulted in more cells with centromere numbers deviating from the mode (Fig. 3D). We also identified that *FOXA1* and *KIF11* upregulation increased the incidence of micronuclei (Fig. 3E). The levels of upregulation achieved for *ACTL6A*, *CENPH*, *EGFR*, *ESPL1*, *OIP5* or *PLK4* did not give rise to CIN and, thus, these genes were not considered further. Hence, our assay identified several genes that can initiate CIN, even when individually upregulated at moderate levels in human diploid cells.

### *KIF11* upregulation results in correctly targeted and increased KIF11 protein levels

We then sought to understand the mechanistic links between activation of specific genes and the CIN phenotypes. We focused on KIF11 because it gave the strongest result in the MN counts, in addition to showing a significant result in mode deviation. KIF11 is a homotetrameric plus-end-directed motor, which crosslinks parallel microtubules and slides antiparallel microtubules apart, drives spindle pole separation, and acts as a force brake (Collins et al., 2014; Cross and McAinsh, 2014; Kapitein et al., 2005; Sawin et al., 1992). Although it is not an oncogene, it plays a multifaceted role in mitosis. Under CRISPRa, *KIF11* was upregulated around fourfold on average (Fig. 3B), leading to a small but significant deviation from the modal chromosome count of control cells (Fig. 3D; 67% for KIF11^CRISPRa^ versus 63% for EV^CRISPRa^; *P*=0.03; permutation test) but a dramatic increase in the number of micronuclei (Fig. 3E; 3 MN/100 cells for KIF11^CRISPRa^ versus 0.5 MN/100 cells for EV^CRISPRa^, *P*<0.0005, two-tailed unpaired *t*-test).

**Fig. 4.**
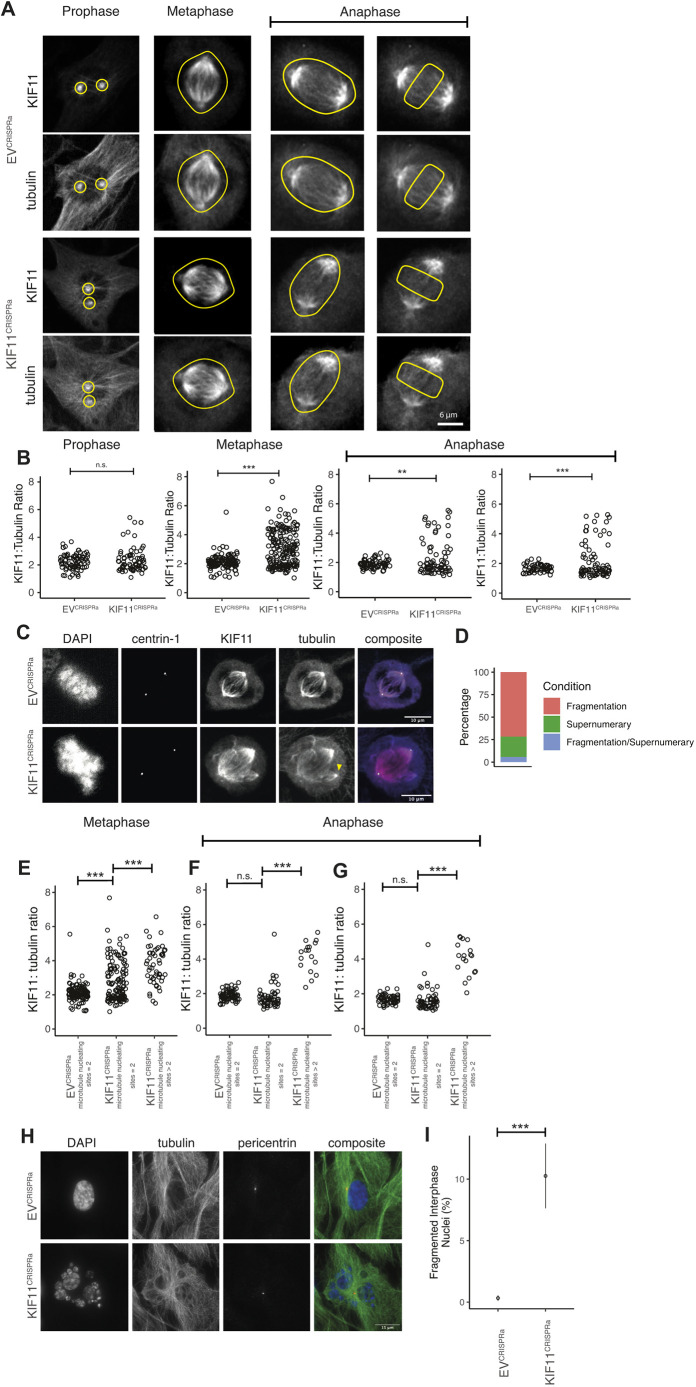
**KIF11^CRISPRa^ increases the levels of KIF11 protein in mitosis, causing PCM fragmentation and mitotic catastrophe.** (A) Prophase, metaphase and anaphase cells stained for KIF11. Yellow ROIs indicate the regions from which protein signals were quantified. (B) KIF11 intensities normalised to those of tubulin for EV^CRISPRa^ and KIF11^CRISPRa^ cells (two-tailed unpaired *t*-test). For prophase cells, the protein signals from the two centrosomes were averaged per cell. For cells in prophase, *n*=85 for EV^CRISPRa^, 77 for KIF11^CRISPRa^, three replicates; metaphase, *n*=145 for EV^CRISPRa^, 140 for KIF11^CRISPRa^, three replicates; anaphase, *n*=74 for EV^CRISPRa^, 116 for KIF11^CRISPRa^, three replicates. (C) Images of EV^CRISPRa^ cells (top) and KIF11^CRISPRa^ cells (bottom) showing abnormal spindle formation upon activation. Left to right: nuclei stained with DAPI, centrioles marked by centrin-1, KIF11 staining, α-tubulin staining and composite of images excluding DAPI. Yellow arrowhead marks the extra microtubule-nucleating site. (D) Phenotypic classification of KIF11^CRISPRa^ cells with >2 microtubule-nucleating sites at metaphase using tubulin staining to count microtubule nucleation and the centrin-1 signal to assess the presence of a centriole. (E) Breakdown of data in B, with KIF11 total spindle intensity normalised against that of tubulin for EV^CRISPRa^ and KIF11^CRISPRa^ cells with two microtubule-nucleating sites, and KIF11^CRISPRa^ cells with more than two microtubule-nucleating sites (two-tailed unpaired *t*-test). (F,G) KIF11 total spindle intensity normalised against that of tubulin for EV^CRISPRa^ and KIF11^CRISPRa^ cells with two microtubule-nucleating sites, and KIF11^CRISPRa^ cells with more than two microtubule-nucleating sites in anaphase cells from the whole-spindle (F) and interpolar (G) microtubules (two-tailed unpaired *t*-test). (H) Images of EV^CRISPRa^ (top) and KIF11^CRISPRa^ (bottom) cells showing fragmented nuclei after activation. Left to right: DAPI, α-tubulin, pericentrin and composite images. (I) Percentage of cells with fragmented nuclei in interphase in EV^CRISPRa^ and KIF11^CRISPRa^ cells (Fisher's exact test, *n*=1351 and 1278 cells for EV^CRISPRa^ and KIF11^CRISPRa^, respectively, three replicates). Data show the mean±s.e.m. n.s., not significant; **P<0.005; ***P<0.0005. Scale bars: 6 μm (A); 10 μm (C); 15 μm (H).

To confirm that increased *KIF11* mRNA resulted in elevated levels of the KIF11 protein on the mitotic spindle, we imaged cells that were stained for KIF11 and tubulin to account for natural cell-to-cell variation. We confirmed that even though the mean fluorescence intensity of tubulin was significantly different between EV^CRISPRa^ and KIF11^CRISPRa^ cells (*P*<0.0005, two-tailed unpaired *t*-test, Fig. S2A), the difference of 6.5% for tubulin was much less than that of KIF11 (38.8%, Fig. S2B). We therefore examined cells using the ratio of the fluorescence intensity of KIF11 to that of tubulin. In prophase cells, we quantified protein levels in regions surrounding the centrosomes and found no significant difference in the amount of KIF11 localised there (Fig. 4A,B; KIF11:tubulin ratio of 2.7 for KIF11^CRISPRa^ versus 2.3 for EV^CRISPRa^, *P*=0.2, two-tailed unpaired *t*-test). We next examined mitotic spindles in metaphase (Fig. 4A,B; 3.0 for KIF11^CRISPRa^ versus 2.1 for EV^CRISPRa^, *P*<0.0005, two-tailed unpaired *t*-test) and anaphase (Fig. 4A,B; 2.3 for KIF11^CRISPRa^ versus 1.9 for EV^CRISPRa^, *P*=0.004, two-tailed unpaired *t*-test) and found a significant increase in the amount of KIF11 localisation, but also a large variability across cells. This was also evident in the increased localisation of KIF11 to interpolar microtubules (Fig. 4A,B; KIF11:tubulin ratio of 2.2 for KIF11^CRISPRa^ versus 1.7 for EV^CRISPRa^, *P*<0.0005, two-tailed unpaired *t*-test). We plotted the density of KIF11 intensity values in metaphase (Fig. S2C), which clearly demonstrates the population for which protein upregulation was successful. Using a threshold for KIF11 levels in EV^CRISPRa^ cells (mean+two standard deviations), we found that 45% of KIF11^CRISPRa^ cells had increased levels of KIF11 at the spindle.

### Increased KIF11 gives rise to pericentriolar material fragmentation and mitotic catastrophe

While quantifying KIF11 protein levels in KIF11^CRISPRa^ cells, we observed that there were more than two sites of microtubule nucleation (Fig. 4C, yellow arrow) suggesting the presence of multiple centrosomes. To investigate whether *KIF11* upregulation causes centrosome amplification, we introduced eGFP-tagged centrin-1 (CETN1, a centriole marker) into the RPE1-MPH cell line and used the RPE1-MPH-eGFP-centrin-1 cell line for *KIF11* upregulation.

Comparing tubulin and centrin-1 images from the same mitotic spindles, we found that there were only two centrosomes with centrin-1 foci, even in the presence of more than two microtubule nucleation sites. This suggested that the multiple microtubule-nucleating sites might be a result of pericentriolar material (PCM) fragmentation. By scoring microtubule nucleation sites as eGFP–centrin-1 positive or negative, we found that 71.7% cells underwent PCM fragmentation (more than two microtubule-nucleating sites and only two that were centrin-1 positive), 22.6% had supernumerary centrosomes (more than two microtubule-nucleating sites and all of them being centrin-1 positive) and 5.7% cells showed both supernumerary centrosomes and PCM fragmentation (Fig. 4D).

To investigate whether PCM fragmentation correlated with elevated KIF11 protein levels, we classified the KIF11 intensities into cells with two or more microtubule-nucleating sites. In KIF11^CRISPRa^ cells, we found that metaphase cells with more than two microtubule-nucleating sites had higher levels of KIF11 compared to the cells with two microtubule-nucleating sites (Fig. 4E; KIF11:tubulin ratio of 3.7 for KIF11^CRISPRa^ cells with >2 microtubule-nucleating sites versus 2.7 for those with two microtubule-nucleating sites, *P*<0.0005, Bonferroni-corrected two-tailed unpaired *t*-test). The same trend was observed in anaphase cells, whether the signal was measured from the whole spindle (Fig. 4F; 4.2 for KIF11^CRISPRa^ cells with >2 microtubule-nucleating sites versus 1.8 for those with two microtubule-nucleating sites, *P*<0.0005, Bonferroni-corrected *t*-test) or the interpolar microtubules (Fig. 4G; 4.0 for KIF11^CRISPRa^ cells with >2 microtubule-nucleating sites versus 1.6 for those with two microtubule-nucleating sites, *P*<0.0005, Bonferroni-corrected two-tailed unpaired *t*-test). When we examined nuclei in KIF11^CRISPRa^ cells, we observed a significant percentage of cells with fragmented interphase nuclei, resembling mitotic catastrophe (Fig. 4H,I; 10.3% in KIF11^CRISPRa^ cells versus 0.3% in EV^CRISPRa^ cells, *P*<0.0005, two-tailed unpaired *t*-test). Our results show that a moderate increase of the KIF11 protein on the mitotic spindle compromises mitosis by giving rise to PCM fragmentation and fragmented interphase nuclei.

### Increased KIF11 impairs chromosome alignment, causes persistent PCM fragmentation and lagging chromosomes, which give rise to micronuclei

**Fig. 5.**
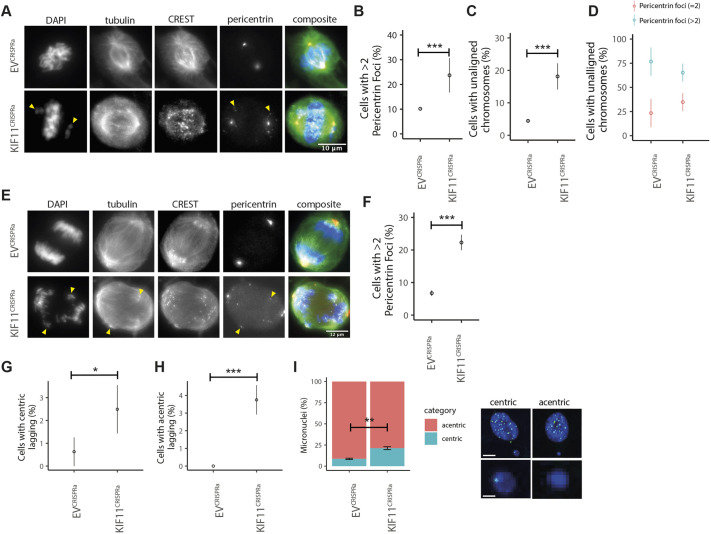
**Increased KIF11 causes PCM fragmentation and unaligned and lagging chromosomes, which give rise to increased numbers of micronuclei.** (A) Images of EV^CRISPRa^ (top) and KIF11^CRISPRa^ (bottom) cells showing dispersed pericentrin and erroneous chromosome congression upon activation. Left to right: DAPI, α-tubulin, CREST, pericentrin and composite images. Yellow arrowheads mark unaligned chromosomes and extra pericentrin foci. (B) Percentage of metaphase cells with more than two pericentrin foci in EV^CRISPRa^ and KIF11^CRISPRa^ cells (Fisher's exact test). (C) Percentage of metaphase cells with unaligned chromosomes in EV^CRISPRa^ and KIF11^CRISPRa^ cells (Fisher's exact test). (D) Percentage of metaphase cells with unaligned chromosomes, grouped by pericentrin foci count of two (red) or greater than two (blue) in EV^CRISPRa^ and KIF11^CRISPRa^ cells (Fisher's exact test). For B–D, *n*=315 and 324 cells for EV^CRISPRa^ and KIF11^CRISPRa^, respectively, three replicates. (E) Images of EV^CRISPRa^ (top) and KIF11^CRISPRa^ (bottom) cells showing highly dispersed pericentrin and erroneous chromosome segregation in activated cells with more than two pericentrin foci. Left to right: DAPI, α-tubulin, CREST, pericentrin and composite images. Yellow arrowheads mark lagging chromosomes and extra pericentrin foci. (F) Percentage of anaphase cells with more than two pericentrin foci in EV^CRISPRa^ and KIF11^CRISPRa^ cells (Fisher's exact test, *n*=330 and 317 cells for EV^CRISPRa^ and KIF11^CRISPRa^, respectively, three replicates). (G,H) Percentage of anaphase cells with CREST-positive (centric) lagging chromosomes (G) and CREST-negative (acentric) lagging chromosomes (H) in EV^CRISPRa^ and KIF11^CRISPRa^ cells (Fisher's exact test). (I) Quantification (left) of acentric and centric micronuclei in EV^CRISPRa^ and KIF11^CRISPRa^ cells (Fisher's exact test) with example composite images (right) of nuclei stained with DAPI and centromeres stained with CREST. The lower panels are magnified views of micronuclei. Data show the mean±s.e.m. *P<0.05; ***P<0.0005. Scale bars: 10 μm (A); 12 μm (E); 6 μm (I, top); 2 μm (I, bottom).

To further understand how increased KIF11 compromised mitosis, we fixed and stained KIF11^CRISPRa^ and EV^CRISPRa^ cells using antibodies to mark the mitotic spindle (α-tubulin), the centromeres (CREST), PCM (pericentrin or PCNT) and DNA (DAPI) (Fig. 5A). We found a significantly larger percentage of cells with more than two pericentrin foci (Fig. 5A, yellow arrows) in metaphase KIF11^CRISPRa^ cells (Fig. 5B; 23.7% for KIF11^CRISPRa^ versus 10.2% for EV^CRISPRa^, *P*<0.0005, Fisher's exact test), consistent with our previous observation of more than two microtubule-nucleating sites without a centriole. These cells exhibited disordered chromosome mass as seen by DAPI staining, indicating that not all chromosomes aligned correctly at the metaphase plate (Fig. 5A, yellow arrows). We found that the proportion of KIF11^CRISPRa^ cells with unaligned chromosomes was over threefold higher than in EV^CRISPRa^ cells (Fig. 5C; 18.0% for KIF11^CRISPRa^ versus 4.0% for EV^CRISPRa^, *P*<0.0005, Fisher's exact test). In both conditions, more than two pericentrin foci often coexisted with the occurrence of unaligned chromosomes (Fig. 5D). To test whether the unaligned chromosomes corresponded to unattached chromosomes, we fixed cells and stained for CENP-A and MAD2, a key checkpoint component that localises on unattached kinetochores (Chen et al., 1998) (Fig. S3A), but we did not find a significant difference between the two conditions (Fig. S3B; 62% of KIF11^CRISPRa^ versus 45% of EV^CRISPRa^ cells with MAD2-positive kinetochores, *P*=0.2, Fisher's exact test).

In anaphase cells, often two bright pericentrin foci were observed at the spindle poles of what resembled a bipolar spindle, however smaller foci were dispersed around the cell and were able to nucleate microtubules (Fig. 5E, small microtubule-nucleating sites, yellow arrows). A significantly higher proportion of KIF11^CRISPRa^ cells exhibited more than two pericentrin foci in anaphase (Fig. 5F; 22.3% of KIF11^CRISPRa^ versus 6.7% of EV^CRISPRa^ cells, *P*<0.0005, Fisher's exact test). Having more than two pericentrin foci was often associated with lagging chromosomes (Fig. 5E, yellow arrows). We scored the number of cells with lagging chromosomes and found a significant increase in the percentage of cells with both centric (Fig. 5G; 2.5% for KIF11^CRISPRa^ cells versus 0.6% for EV^CRISPRa^ cells, *P*<0.0005, Fisher's exact test) and acentric (Fig. 5H; 3.7% for KIF11^CRISPRa^ cells versus 0% EV^CRISPRa^ cells, *P*<0.0005, Fisher's exact test) lagging chromosomes. To further investigate these results, we stained KIF11^CRISPRa^ cells for CENP-A and DAPI and scored micronuclei with centric (CENP-A-positive) or acentric chromosomes (CENP-A-negative). We found a significant increase in the proportion of micronuclei with centric chromosomes in KIF11^CRISPRa^ cells (21% for KIF11^CRISPRa^ versus 8% for EV^CRISPRa^, *P*=0.004, Fisher's exact test), suggesting *KIF11* upregulation increases the number of centric lagging chromosomes. In both conditions, most of the micronuclei were CENP-A negative (Fig. 5I).

Our results show that increased levels of KIF11 result in PCM fragmentation, which impairs chromosome alignment, persists in anaphase and increases the incidence of lagging chromosomes that remain unresolved and give rise to micronuclei.

### Elevated localisation of KIF11 to the mitotic spindle leads to chromosome segregation failures

**Fig. 6.**
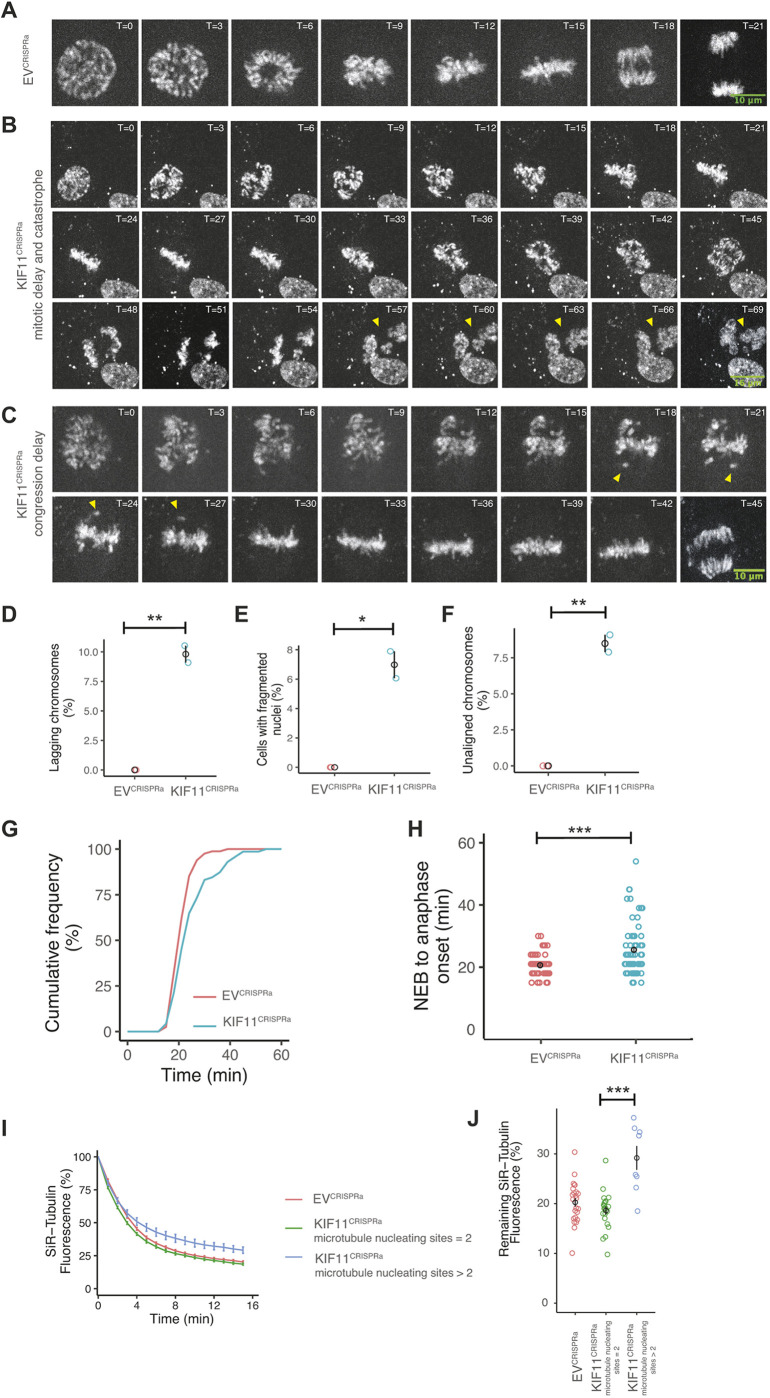
**Live imaging confirms that increased KIF11 levels lead to error-prone chromosome segregation.** (A–C) Images from live imaging of cells stained with SiR-DNA undergoing normal mitosis (A) delayed mitosis and catastrophe (yellow arrowheads) (B), and delayed chromosome congression (yellow arrowheads) (C). Timepoints (T) are indicated in minutes. (D) Percentage of cells with non-resolved lagging chromosomes that were not associated with a subsequent mitotic catastrophe in EV^CRISPRa^ and KIF11^CRISPRa^ cells from two independent experiments (Fisher's exact test). Black circles represent the average for each condition, error bars show the s.e.m. (E) Percentage of cells with mitotic catastrophe in EV^CRISPRa^ and KIF11^CRISPRa^ cells (Fisher's exact test). (F) Percentage of cells experiencing delayed chromosome congression in EV^CRISPRa^ and KIF11^CRISPRa^ cells (Fisher's exact test). For D–F, *n*=330 and 317 cells for EV^CRISPRa^ and KIF11^CRISPRa^, respectively, three replicates. (G) Cumulative frequency of the time from NEB to anaphase onset of EV^CRISPRa^ and KIF11^CRISPRa^ cells progressing through mitosis. (H) Quantification of the time from NEB to anaphase onset of EV^CRISPRa^ and KIF11^CRISPRa^ cells (two-tailed unpaired *t*-test). For G,H, *n*=81 and 71 cells for EV^CRISPRa^ and KIF11^CRISPRa^, respectively, two replicates. (I) Quantification of the reduction of SiR-tubulin fluorescence signal as a percentage of the initial intensity value at the start of the time lapse (T=0 min) for EV^CRISPRa^ and KIF11^CRISPRa^ cells with 2 microtubule-nucleating sites or with more than 2 microtubule-nucleating sites. Cells were synchronised with MG132 during SiR-tubulin incubation and then treated with nocodazole at T=0. Curves fitted using a two-phase decay model plotted with s.e.m. (J) Percentage of the remaining SiR-Tubulin fluorescence at the end of the time-lapse (T=15 min) for EV^CRISPRa^ and KIF11^CRISPRa^ cells with two microtubule-nucleating sites or with more than two microtubule-nucleating sites (two-tailed unpaired *t*-test). For I,J, *n*=24, 22 and 8 cells for EV^CRISPRa^ cells, KIF11^CRISPRa^ bipolar cells and KIF11^CRISPRa^ multipolar, respectively. Data show the mean±s.e.m. *P<0.05; **P<0.005; ***P<0.0005. Scale bars: 10 μm (A,C); 16 μm (B).

To investigate the mitotic defects observed in fixed KIF11^CRISPRa^ cells, we performed live-cell imaging of EV^CRISPRa^ and KIF11^CRISPRa^ cells using SiR-DNA, a fluorescent DNA probe (Fig. 6A). We identified fragmented nuclei (Fig. 6B) as well as delayed congression (Fig. 6C). We found that a small percentage of KIF11^CRISPRa^ cells had unresolved lagging chromosomes (Fig. 6D; 9.8% KIF11^CRISPRa^ cells, *P*=0.006, Fisher's exact test), whereas a significant percentage underwent nuclear fragmentation (Fig. 6E; 7.0% KIF11^CRISPRa^ cells, *P*=0.02, Fisher's exact test). The percentage of cells displaying delayed chromosome congression was also elevated (Fig. 6F; 8.5% KIF11^CRISPRa^ cells, *P*=0.01, Fisher's exact test). We timed the duration of mitosis from nuclear envelope breakdown (NEB) to the onset of anaphase (Fig. 6G), finding a 3 min median delay in anaphase onset for KIF11^CRISPRa^ cells compared to EV^CRISPRa^ cells, but a more significant delay on the order of 10 min affecting the slower tail population of KIF11^CRISPRa^ cells (Fig. 6H; 25.6 min for KIF11^CRISPRa^ cells versus 20.6 min for EV^CRISPRa^ cells, *P*<0.0005, two-tailed unpaired *t*-test).

To investigate the timing of PCM fragmentation, we performed overnight live imaging of RPE1-MPH-eGFP-centrin-1 KIF11^CRISPRa^ cells with SiR-tubulin, a fluorescent tubulin marker. We identified cells with PCM fragmentation, based on the presence of eGFP-centrin-1-negative tubulin asters (Fig. S3C, yellow arrows). Using eGFP–centrin-1 background fluorescence to identify NEB, we scored the degree (number of asters) and timing of PCM fragmentation with respect to NEB (example trajectories are shown in Fig. S3D). The degree of fragmentation varied, with some cells developing ten tubulin asters and some clustering one to two of their PCM fragments. PCM fragmentation was not observed until after NEB, taking an average of 10 min to establish (Fig. S3E). This experiment also provided a direct link between PCM fragmentation and fragmented interphase nuclei in the daughter cells.

To investigate cell fate, we imaged KIF11^CRISPRa^ cells using SiR-DNA and differential interference contrast (DIC) microscopy for 60 h (Fig. S4A). During the first division (mother cells), 83.1% of cells divided correctly, 14% exhibited nuclear fragmentation, 2.2% had lagging chromosomes and only one cell underwent multipolar cell division (Fig. S4B). During the second division, 84.4% of the daughter cells arising from mother cells that divided normally went on to divide normally, 2.9% arrested and 1.2% had a lagging chromosome. Most of the cells that exhibited fragmented nuclei in the first division arrested (77.4%) or died (19.4%) (inferred by the irregular cytoplasmic morphology and persistent DNA condensation; Fig. S4B). We stained EV^CRISPRa^ and KIF11^CRISPRa^ cells for p53 and found that 75.7% of cells with fragmented interphase nuclei had elevated p53 staining and underwent a p53-dependent arrest (Fig. S4C,D). None of the KIF11^CRISPRa^ mother cells with lagging chromosomes gave rise to daughter cells that experienced fragmented interphase nuclei, and the same was true in reverse, suggesting that the two phenotypes occur mutually exclusively.

To investigate the cause of lagging chromosomes, we treated cells with nocodazole and measured the loss of SiR-tubulin fluorescence as the mitotic spindle depolymerised (Wilhelm et al., 2019). This gave a characteristic exponential decay in the levels of SiR-tubulin fluorescence in EV^CRISPRa^ cells and the KIF11^CRISPRa^ cells with two microtubule-nucleating sites (Fig. 6I). However, KIF11^CRISPRa^ cells with more than two microtubule-nucleating sites had significantly more microtubules remaining after 15 min (Fig. 6I,J; the remaining SiR-tubulin levels were 29% in KIF11^CRISPRa^ cells containing >2 tubulin microtubule-nucleating sites and 19% in KIF11^CRISPRa^ cells containing two microtubule-nucleating sites versus 20% in EV^CRISPRa^ cells, *P*=0.2 and *P*=0.003, respectively, two-tailed unpaired *t*-test), demonstrating a correlation between KIF11^CRISPRa^ cells and enhanced microtubule stability. Taken together with data linking higher microtubule stability to lagging chromosomes (Laughney et al., 2015), our results suggest that *KIF11* upregulation causes lagging chromosomes by stabilising microtubules.

### *HSET* upregulation rescues PCM fragmentation induced by *KIF11* upregulation

**Fig. 7.**
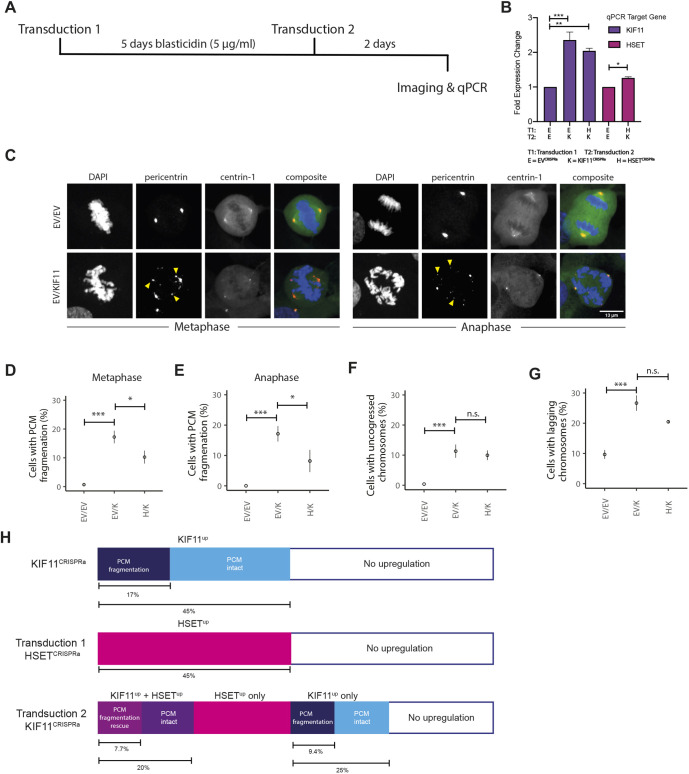
**Multiplex CRIPSR activation of *HSET* and *KIF11* rescues PCM fragmentation and fragmentation-dependent lagging chromosomes.** (A) Schematic outlining the optimised experimental protocol for simultaneous upregulation of *HSET* and *KIF11*. (B) Fold upregulation measured by RT-qPCR after CRISPR co-activation of *HSET* and *KIF11*. (C) Images of normal cell division in EV^CRISPRa^/EV^CRISPRa^ cells (top) and of EV^CRISPRa^/KIF11^CRISPRa^ cells with PCM fragmentation (bottom) in metaphase and anaphase. Left to right: DAPI, pericentrin, centrin-1 and composite images. Yellow arrowheads indicate extra pericentrin foci. Scale bar: 13 μm. (D,E) Percentage of cells with PCM fragmentation after co-activation of HSET and KIF11 in metaphase (D) (*n*=309, 305 and 341 cells for EV^CRISPRa^/EV^CRISPRa^, EV^CRISPRa^/KIF11^CRISPRa^ and HSET^CRISPRa^/KIF11^CRISPRa^, respectively, three replicates) and anaphase (E) (Fisher's exact test). (F) Percentage of cells with uncongressed chromosomes after co-activation of *HSET* and *KIF11* in metaphase (Fisher's exact test). *n*=407, 287 and 341 cells for EV^CRISPRa^/EV^CRISPRa^, EV^CRISPRa^/KIF11^CRISPRa^, HSET^CRISPRa^/KIF11^CRISPRa^, respectively, three replicates. (G) Percentage of cells with lagging chromosomes after co-activation of *HSET* and *KIF11* (Fisher's exact test). For E,G, *n*=292, 221 and 205 cells for EV^CRISPRa^/EV^CRISPRa^, EV^CRISPRa^/KIF11^CRISPRa^ and HSET^CRISPRa^/KIF11^CRISPRa^, respectively, three replicates. Data show the mean±s.e.m. n.s., not significant; *P<0.05; ***P<0.0005. (H) Model of phenotypic outcomes due to *KIF11* single activation (top) and *HSET*/*KIF11* co-activation (middle and bottom). Rectangles indicate different cell subpopulations with phenotype, if any, as indicated. Percentages of total population are shown below each bar.

Upregulating *KIF11* could disrupt opposing forces within the mitotic spindle, leading to PCM fragmentation and defective mitosis, as excess KIF11 could generate unbalanced outward pushing forces on antiparallel microtubules (Cross and McAinsh, 2014). To investigate this, we used CRISPRa co-activation for the simultaneous upregulation of *KIF11* and *HSET*, which encodes a minus-end directed motor that counterbalances KIF11 activity, crosslinks and slides antiparallel microtubules, and promotes centrosome clustering (Fink et al., 2009; Kapitein et al., 2005; Mountain et al., 1999). Increased levels of HSET on the spindle should increase the inward pulling forces on antiparallel microtubules, balancing increased levels of KIF11. We first transduced RPE1-MPH-eGFP-centrin-1 cells with a guide RNA (gRNA) targeting *HSET* (transduction 1) followed by 5 days of antibiotic selection. We then transduced these cells with a gRNA targeting *KIF11* (transduction 2), before harvesting samples and imaging 48 h later (Fig. 7A). The protocol resulted in 1.26- and 2-fold increases in gene expression for *HSET* and *KIF11*, respectively, relative to cells transduced twice with EV^CRISPRa^ (EV^CRISPRa^/EV^CRISPRa^ cells) (Fig. 7B; *P*<0.05 and *P*<0.01, respectively, two-tailed unpaired *t*-test). Despite this difference, the level of *KIF11* upregulation was similar between EV^CRISPRa^/KIF11^CRISPRa^ cells and HSET^CRISPRa^/KIF11^CRISPRa^ cells, enabling investigation of whether the small degree of *HSET* upregulation could rescue PCM fragmentation.

We fixed and stained cells with pericentrin to identify PCM fragmentation in both metaphase and anaphase cells (Fig. 7C). We found an increased presence of fragmentation in EV^CRISPRa^/KIF11^CRISPRa^ cells in both metaphase (Fig. 7D; 17.2% cells had PCM fragmentation for EV^CRISPRa^/KIF11^CRISPRa^ versus 0.7% for EV^CRISPRa^/EV^CRISPRa^, *P*<0.0001, Fisher's exact test) and anaphase (Fig. 7E; 17.1% for EV^CRISPRa^/KIF11^CRISPRa^ versus 0% for EV^CRISPRa^/EV^CRISPRa^, *P*<0.0001, Fisher's exact test). In HSET^CRISPRa^/KIF11^CRISPRa^ cells, we observed a significant reduction in the percentage of cells with PCM fragmentation compared to EV^CRISPRa^/KIF11^CRISPRa^ cells, in both metaphase (Fig. 7D; 10.3% for HSET^CRISPRa^/KIF11^CRISPRa^ cells, *P*=0.04, Fisher's exact test) and anaphase (Fig. 7E; 8.2% for HSET^CRISPRa^/KIF11^CRISPRa^ cells, *P*=0.02, Fisher's exact test). However, *HSET* upregulation did not change the prevalence of cells with uncongressed chromosomes (Fig. 7F; 11.3% cells had uncongressed chromosomes for EV^CRISPRa^/KIF11^CRISPRa^ versus 0.4% for EV^CRISPRa^/EV^CRISPRa^, *P*<0.0001; 10.0% for HSET^CRISPRa^/KIF11^CRISPRa^ versus 11.3% for EV^CRISPRa^/KIF11^CRISPRa^ *P*=0.1, Fisher's exact test). We also did not observe a significant reduction in the occurrence of lagging chromosomes overall (Fig. 7G; 26.7% cells had lagging chromosomes for EV^CRISPRa^/KIF11^CRISPRa^ versus 10.0% for EV^CRISPRa^/EV^CRISPRa^, *P*<0.0001; 20.5% for HSET^CRISPRa^/KIF11^CRISPRa^ versus 26.7% for EV^CRISPRa^/KIF11^CRISPRa^ *P*=0.3, Fisher's exact test).

At first glance, it appeared that the rescue of PCM fragmentation by co-activating *HSET* with *KIF11* was only partial (Fig. 7D,E). However, we previously estimated *KIF11* activation efficiency at the cell level to be approximately 45%, i.e. KIF11 was upregulated in 45% of all transduced cells (KIF11^up^ cells) (Fig. S2), and a fraction of these cells (representing 17% of the total population) had PCM fragmentation. If we assume that *HSET* activation was similarly efficient (i.e. 45% of the total cell population were HSET^up^ cells) and whether a given cell had upregulated *KIF11* (KIF11^up^) or upregulated *HSET* (HSET^up^) occurred independently, then only 20% of the total cells would have been both KIF11^up^ and HSET^up^ (45%×45%). The rate of KIF11^up^-induced PCM fragmentation would have yielded 7.7% of cells that were both doubly activated and PCM fragmented (Fig. 7H). It is this population that could be rescued and, indeed, we saw an approximately 8% drop in PCM-fragmented KIF11^up^/HSET^up^ cells in Fig. 7D,E. If these assumptions hold true, then our results imply a complete rescue. In any case, whether the rescue was complete or not, it is clear that the PCM fragmentation was caused by excess KIF11 that was insufficiently opposed by endogenous HSET activity.

## DISCUSSION

We have developed a high-throughput, single-cell, image-based pipeline to detect CIN initiation that occurs downstream of moderate upregulation of single genes in normal cells. Detecting all centromeres in thousands of cells is advantageous as different chromosomes mis-segregate at different rates (Worrall et al., 2018). Techniques which detect few chromosomes, such as FISH, underestimate aneuploidy. Moreover, the use of proxy chromosomes, e.g. human artificial chromosomes (Harrington et al., 1997), is unable to indicate the true extent of aneuploidy in endogenous chromosomes. Furthermore, methods that measure DNA content, such as fluorescence-activated cell sorting (FACS) (Doležel et al., 2012), have low resolution due to varying chromosome sizes. Our high-throughput confocal-image-based algorithm achieves high sensitivity and low mean absolute error. It could be adapted to count or detect other objects (e.g. centrosomes or centrioles) or point markers (e.g. γ-H2AX), and, combined with staining of the cell membrane, allow segmentation of multinucleated cells and link micronuclei to specific cells. The software already quantifies fluorescence, enabling protein localisation and expression studies. Moreover, it is adaptable to live-cell imaging, enabling large-scale mechanistic and cell-fate studies, and can be extended for multiplex gene activation or scaled up for genome-wide studies using CRISPR-activation guide, knockdown or drug libraries.

Moderate upregulation of several genes produced detectable aneuploidies, with the results from FWHM and deviation from mode measurements largely in agreement. The biggest shifts in centromere counts were seen in upregulation of *CCND1*, *FOXA1* and *NEK2*. Previously, overexpression of *CCND1* in mouse embryonic fibroblasts (MEFs) increased the expression of genes involved in chromatin reorganisation and chromosome segregation, increasing the incidence of 4N/8N cells, and chromosomal translocations (Casimiro and Pestell, 2015). Similarly, overexpression of NEK2 resulted in centrosome amplification and binucleation in MCF10A cells (Rivera-Rivera et al., 2021). Previously, 500- to 10,000-fold overexpression of MAD2 was found to induce tetraploidy and aneuploidy (Sotillo et al., 2007). Here, we have shown that gene activation inducing an average fivefold upregulation in gene expression can trigger aneuploidy. We found that upregulation of *KIF11*, *NEK2*, *FOXA1* and *PIK3CA* skewed centromere counts toward 3N cells, supportive of a proposed stable triploid state (Laughney et al., 2015). The finding that a 20% upregulation in *PIK3CA* expression resulted in aneuploidy but did not increase the number of MN suggests that a moderate increase in the levels of PIK3CA compromises genome integrity without micronuclei formation, in agreement with previous findings that PIK3CA^H1047R^ did not induce chromosome segregation errors in MEFs (Berenjeno et al., 2017).

Although KIF11 inhibition has been the focus of anti-mitotic drugs, its upregulation has not been considered in previous mechanistic studies. Modest upregulation of *KIF11* increased micronuclei incidence but resulted in only a small but significant modal deviation of chromosome count. This can be explained by the fact that only 45% of KIF11^CRISPRa^ cells are KIF11^up^. KIF11 increase resulted in PCM fragmentation and lagging chromosomes, suggestive of a KIF11-dependent fragility of the mitotic spindle. One possibility is that a threshold exists at which the force imbalance between the elevated KIF11 and opposing mitotic motors becomes too great. HSET opposes KIF11 activity in crosslinking and sliding antiparallel microtubules (Mountain et al., 1999), and we did find a reduction in PCM fragmentation in HSET^CRISPRa^/KIF11^CRISPRa^ cells. KIF11 also antagonises the activity of the dynein-dynactin-NuMA complex, which acts to cluster microtubule ends in mitosis. Our observation of multiple PCM fragments that nucleate microtubules and interfere with chromosome congression resonates with previous findings that dynein or NuMA knockouts result in unstable mitotic spindles, which are restored upon KIF11 inhibition (Hueschen et al., 2019). PCM fragmentation occurred after NEB and was dependent on spindle formation. Interestingly, KIF11-dependent fragmentation of acentriolar microtubule-organising centres post NEB is essential for accurate spindle assembly in mouse oocyte spindle assembly (Clift and Schuh, 2015).

Multipolar spindle geometries, even if transient, facilitate kinetochore merotelic attachments and lagging chromosomes (Silkworth and Cimini, 2012). Although co-activation of *KIF11* and *HSET* reduced the number of cells with PCM fragmentation, it did not significantly reduce the frequency of lagging chromosomes, suggesting that increased KIF11 compromised the mitotic spindle in a way that is not rescued by the increase in HSET. *KIF11* was upregulated at different levels in our experiments, with the higher levels of KIF11 correlating with PCM fragmentation. These cells also exhibited higher microtubule stability, which could be explained by enhanced crosslinking between microtubules emanating from the multiple PCM fragments, giving rise to lagging chromosomes.

Although centrosome abnormalities are frequently seen in solid tumours, there are no reports of spindle pole fragmentation caused by overexpression of KIF11. Most studies use KIF11 overexpression only as a prognostic marker in cancer, correlating with poor patient survival (Daigo et al., 2018; Jiang et al., 2017; Venere et al., 2015; Zhou et al., 2021). Only the cells with *KIF11* upregulation to higher levels exhibited PCM fragmentation, which often led to mitotic catastrophe, whereas cells with milder upregulation of *KIF11* presented frequent lagging chromosomes but otherwise normal spindles, allowing the cells to divide. This demonstrates how subtle changes in protein levels can lead to very different outcomes. The former case of cells that undergo mitotic catastrophe is less consequential as no progeny remain, whereas in the latter case, cells propagate with aneuploid karyotypes, enabling a route to CIN. These results show that *KIF11* upregulation to a relatively moderate magnitude is sufficient to initiate CIN.

## MATERIALS AND METHODS

### Cell culture, virus production and generation of stable cell lines

RPE1 cells were obtained from American Type Culture Collection and maintained in Dulbeccos's modified Eagle medium F12 (DMEM/F12, Sigma-Aldrich), supplemented with 10% fetal bovine serum (Sigma-Aldrich), 2 mM L-glutamine (Gibco), and 1× penicillin/streptomycin (Gibco). HEK293FT cells were obtained from Thermo Fisher Scientific and maintained in DMEM (high glucose, pyruvate, Sigma-Aldrich), supplemented with 10% fetal bovine serum, 2 mM L-glutamine, 1× penicillin/streptomycin, 0.1 mM MEM Non-Essential Amino Acids (Sigma-Aldrich), and 0.5 mg/ml geneticin (Gibco). Virus production was performed in HEK293FT cells using the ViraPower Lentiviral Expression System (Thermo Fisher Scientific) according to the manufacturer's protocol. Lentiviral titres were calculated using the colony formation assay and the values were subsequently confirmed with Lenti-X GoStix (Takara Bio) measurements. To generate a clonal RPE1-MPH cell line, RPE1 cells were transduced with a lentivirus expressing pLenti-MPHv2 (Addgene plasmid #89308; deposited by Feng Zhang, Massachusetts Institute of Technology, USA) modified to incorporate the NeoR gene. Single colonies were selected using 0.3 mg/ml geneticin. To generate an RPE1-MPH-eGFP-centrin-1 cell line, eGFP–centrin-1 (a gift from Andrew McAinsh, University of Warwick, UK) was cloned into the pLVX-puro vector (Takara Bio) and RPE1 cells were transduced with the resulting lentivirus. FACS was used to isolate a bulk population positive for transgene expression. To generate the RPE1-p53-shRNA cells, RPE1 cells were transduced with shp53 pLKO.1 puro (Addgene plasmid #19119; deposited by Bob Weinberg, Massachusetts Institute of Technology, USA). To generate a stable cell line expressing miRFP709–Cdt1(1/100), miRFP709–Cdt1(1/100) was subcloned from pCSII-EF-miRFP709-hCdt(1/100) (Addgene plasmid #80007; deposited by Vladislav Verkhusha, Albert Einstein College of Medicine, USA) into pLVX-puro, and the RPE1-MPH cell line was subsequently transduced with viral particles containing the final construct and selected with 10 μM nutlin-3 (Roche) for functional loss of p53. All cell lines were tested for mycoplasma contamination and confirmed to be mycoplasma free.

### Drug treatments and antibodies

Inhibitors were dissolved in DMSO and were used at the following concentrations: 50 nM CENP-E inhibitor (GSK923295, Cambridge Bioscience), 2–3 μM MPS1 inhibitor (NMS-P715, Merck Chemicals) and 0.15–0.45 μM reversine (Sigma-Aldrich).

Antibodies were used as follows: mouse α-tubulin (Thermo Fisher Scientific, 32-2500), 1:5000 (western blotting or WB) and 1:500 (immunofluorescence or IF); rabbit α-tubulin (Abcam, ab52866), 1:500 (IF); mouse CENP-A (Abcam, ab13939), 1:200 (IF); human CREST (Antibodies Incorporated, 15-234-0001), 1:300 (IF); rabbit pericentrin (Abcam, ab4448), 1:2000 (IF); rabbit KIF11 (Novus Biologicals, NB5000-181), 1:1000 (IF); mouse p53 (Cell Signaling Technologies, 48818S), 1:1000 (IF); and rabbit MAD2 (Millipore UK, MABE866), 1:1000 (WB) and 1:500 (IF).

### CRISPRa using the Synergistic Activation Mediators System

CRISPR activation was performed according to the protocol detailed by Joung et al. (2017). Up to eight gRNAs targeting the promoter of each gene of interest were designed using the gRNA design tool available from the Zhang Lab SAM website (https://zlab.bio/guide-design-resources), and cloned into pLenti-SAM v2 (Addgene plasmid #75112; deposited by Feng Zhang, Massachusetts Institute of Technology, USA) using a standard restriction enzyme gRNA cloning protocol (https://www.addgene.org/crispr/zhang/). To perform CRISPR gene activation using the SAM system, RPE1-MPH cells were transduced with lentivirus expressing the pLenti-SAM v2 construct (containing dCas9 and a single gRNA). The RPE1-MPH-SAM cells were maintained with blasticidin (Thermo Fisher Scientific) selection and processed further 5–7 h post transduction.

### RT-qPCR

Gene expression was measured by RT-qPCR at least 48 h post transduction. Extraction of RNA was performed using QIAGEN Mini or Macro RNeasy kits (depending on sample size), with on-column digestion of DNA using the QIAGEN DNase kit. RNA concentration was quantified using a NanoDrop (Thermo Fisher Scientific). cDNA synthesis was performed with 500 ng RNA, using SuperScript IV VILO Master Mix (Thermo Fisher Scientific) according to manufacturer instructions. RT-qPCR was performed according to manufacturer instructions using Power SYBR Green Master Mix (Applied Biosystems), Quantitect primers (QIAGEN) specific to the target gene and 1 μl cDNA as a template. Experiments were performed in an Eppendorf Realplex 4 thermocycler. The ΔΔCT method was used to quantify relative gene expression levels, normalised to the house-keeping gene *GAPDH*.

### Western blotting

Protein expression was measured at least 48 h post transduction. Cells were collected by centrifugation, and cell pellets were washed in PBS and lysed in mammalian protein extraction reagent M-PER (Thermo Fisher Scientific) supplemented with Halt protease and phosphatase inhibitor cocktail (Thermo Fisher Scientific). Protein concentration was determined using the Direct Detect assay (Millipore). Protein samples were run on 4–15% Mini-PROTEAN TGX gels (Bio-Rad) and transferred to nitrocellulose membranes (Thermo Fisher Scientific). Membranes were blocked with 5% milk in TBS containing Tween-20 (TBS-T, Thermo Fisher Scientific) and incubated with primary antibodies diluted in 5% milk in TBS-T overnight at 4°C. For chemiluminescent visualisation, membranes were incubated with horseradish peroxidase-conjugated secondary antibodies (1:5000, GE Healthcare). Protein bands were visualised using the SuperSignal West Pico PLUS Chemiluminescent Substrate (Thermo Fisher Scientific) and an ImageQuant LAS 4000 image analyser (GE Healthcare). For fluorescence visualisation, membranes were incubated with IRDye 680RD or IRDye 800CW secondary antibody (Li-COR), and imaged using an Odyssey CLx system (Li-COR).

### Immunofluorescence

Cells were grown on 22×22 mm cover slips in six-well plates. Coverslips were washed in PBS and fixed in either methanol for staining the spindle, or PTEMF buffer (20 mM PIPES pH 6.8, 10 mM EGTA, 1 mM MgCl_2_, 0.2% Triton X-100, 4% formaldehyde) for staining centromeres and/or kinetochores. Coverslips were then washed in PBS and blocked in 3% bovine serum albumin (BSA) for 1 h at room temperature (RT). Primary antibodies were diluted in 3% BSA, and 100 ml of the solution was pipetted onto parafilm per coverslip. Coverslips were then transferred on top of the antibody solution cell-side down and incubated for 1 h at RT. Coverslips were then transferred back to the six-well plate and washed three times with PBS for 30 min. Secondary antibody incubation was performed using the same method, followed by three PBS washes for 30 min. Coverslips were stained with 1 mg/ml DAPI for 5 min, washed twice with PBS, left to air dry in the dark, and mounted on glass slides in DAKO mounting medium (Agilent). The Click-iT EdU Cell Proliferation Alexa 647 kit (Thermo Fisher Scientific) was used to identify S phase cells. Cells were incubated in culture medium containing 10 mM EdU for 20 min. EdU staining was subsequently performed according to manufacturer's instructions.

### Metaphase spreads

Cells were treated with 100 ng/ml nocodazole (Tocris Bioscience) for 16 h, and metaphase-arrested cells were harvested by mitotic shake off. All centrifugation steps were performed at 300 ***g*** for 8 min. Cells were centrifuged and resuspended in 5 ml hypotonic solution (75 mM KCl) for 25 min at 37°C. Cells were then centrifuged and resuspended in Carnoy's fixative (3:1 methanol:acetic acid), and incubated at RT for 15–30 min. Cells were centrifuged and resuspended in Carnoy's fixative a second time, and incubated at RT for 15–30 min. Cells were centrifuged and resuspended in 50–200 ml Carnoy's fixative. 12 ml of the cell solution was dropped from a height of 1 cm onto a glass slide, which was immediately placed in a covered 50°C water bath for 2 min for drying in a humidified environment to improve chromosome spreading (Deng et al., 2003). Once dry, slides were stained with 1 mg/ml DAPI and a coverslip mounted using DAKO mounting medium.

### Fixed-cell imaging

To quantify mitotic errors and protein levels in mitosis, fixed cells were imaged using a spinning disk confocal microscope (3i), acquiring 30 *z*-stacks of 0.2 mm using a 63×1.4 NA oil immersion objective. When investigating KIF11 spindle localisation, cells were stained for KIF11, α-tubulin and DAPI. Images were analysed in Fiji and various regions of interest (ROIs) were drawn, and the mean intensity values recorded for KIF11 and α-tubulin. To investigate p53 expression, cells were stained for p53 and DAPI, and imaged using a spinning disk confocal microscope (3i), acquiring single-plane images using a 40×1.3 NA objective. These images were analysed in Slidebook, using the inbuilt tools to perform background subtraction, segment nuclei, filter nuclei and measure p53 mean signal intensity. For high content screening of centromeres and micronuclei, cells were grown, stained and imaged in 96-well UltraCarrier plates (Perkin Elmer), using an Opera Phenix HCS imaging system (Perkin Elmer). Images were acquired using a 63×1.15 NA water immersion objective capturing 17 0.5 μm-thick *z*-stacks. Channels were imaged consecutively to minimise bleedthrough.

### Live-cell imaging

All live-cell imaging was performed on a spinning disk confocal microscope (3i), fitted with an environmental chamber maintained at 37°C and supplied with 5% CO_2_. For live-cell imaging, cells were incubated with SiR-DNA or SiR-tubulin (Spirochrome) to a final concentration of 100 nM for 3 h prior to imaging. *Z*-stacks of seven planes with a 2 μm step size were acquired using a 63×1.4 NA oil immersion objective. To determine mitotic errors and timings, cells were imaged overnight (12 h). For the cell-fate experiments, cells were imaged for 60 h.

### Microtubule depolymerisation assay

Cells were incubated with 100 nM SiR-Tubulin for 4 h before imaging. 30 min before imaging, cell culture medium containing 100 nM SiR-tubulin and 10 mM MG132 (Tocris Bioscience) was added to prevent cells from transitioning to anaphase. Metaphase cells were identified using either DIC microscopy or by imaging eGFP-centrin-1, and, immediately before imaging, the medium was removed and replaced with medium containing 100 nM SiR-Tubulin, 10 mM MG132 and 200 ng/ml nocodazole. A *z*-stack of 28 planes with a 2 mm step size, taking an image every 1 min using a 100×1.46 numerical aperture oil objective, was acquired. Cells were imaged for 15 min. Movies were analysed in Fiji by generating a sum intensity projection to extract total intensity within a ROI over time.

### Automated nuclei segmentation

Neural networks require training to learn how to segment images; thus, we prepared 60 fields with manually segmented nuclei and used data augmentation techniques to extract maximum use of these. The output of the model is a prediction probability for whether each pixel is within a nucleus or not. We converted this output to a binary image by thresholding using a 0.5 cutoff.

For nuclei segmentation, we built a convolutional deep neural network (CNN) architecture based on U-Net (Ronneberger et al., 2015) with minor modifications (reduced number of filters on entry layers of each stage of the contracting path) in MATLAB using the Deep Learning Toolbox. Training the network requires a large number of example images with manually segmented nuclei from which to learn, which are time consuming to create. We therefore took a two-step approach. First, we created a MATLAB graphical user interface (GUI) segmentation labeller tool enabling researchers to mark cell nuclei. Use of the tool comprised two steps per nuclei in each image. The first step involved drawing a bounding region, either rectangular or freehand, which might encompass one or more nuclei. This region could also be fine-tuned by adding parts or deleting parts. The second step involved adjusting a fixed or adaptive threshold to capture the majority of nuclei pixels within the region, independently from the rest of the image. The regions defined by the pixels above the threshold were subjected to hole-filling and opening filters to smooth noise, and were then filled to become convex. The label was then stored as a pixel mask together with the image file. This software also allows multi-class labelling (e.g. mitosis, micronuclei); however, in this instance, we only used nuclei segments.

Using the segmentation labeller tool, we labelled nuclei from 57 fields of 2048×2048 pixels, resulting in 1069 example nuclei. We split the labelled images into 51 training and six validation fields. To maximise the utility of this dataset, during every training epoch, we extracted 64 128×128 pixel images, randomly located patches for every field, and applied a series of data-augmentation techniques (reflection, scaling and translation) to significantly increase the effective size of the training dataset. Training was performed on a workstation with dual NVIDIA Quadro P2000 GPUs and a 12-core Intel Xeon 5118 2.30 GHz CPU, and the model achieved a 0.833 weighted intersection over union (IoU) score, comparable with the original U-Net experiments (Ronneberger et al., 2015). After training the segmentation model, we verified its generalisability by applying it on a held-out dataset of nine manually labelled fields, yielding 0.827 weighted IoU. The output of the model is a prediction for whether each pixel is nuclei or not, which we converted to a binary image by thresholding using a 0.5 cutoff. The resulting segmentation was post-processed to remove stray pixels or holes by region filling prior to computing image statistics.

### Segment classification

To eliminate fields not containing any cells, we used an image entropy filter to eliminate low-entropy fields, based on the observation that fields with cells had significantly higher image entropy. Prior to further analysis, we applied an EdU intensity threshold to exclude nuclei in S phase. To set the EdU threshold, we generated a stable cell line expressing fluorescently tagged Cdt1(1/100), a truncated version of the G1 marker from the FUCCI system, which is used to label the different stages of the cell cycle without influencing cell cycle progression (Shaner et al., 2013; Zielke and Edgar, 2015). By comparing the intensity of EdU and miRFP709-Cdt1(1/100) from single nuclei, we identified an EdU intensity level which excluded >99% Cdt1 positive cells. We chose EdU over Cdt1 based on the previously reported involvement of Cdt1 overexpression in CIN and DNA damage (Tatsumi et al., 2006). Inspecting the resulting segments, we found that in some cases, large detritus were occasionally partially classified as nuclei. Occasionally, nuclei found in proximity were not resolved as two distinct segments, but classified as a large single nucleus. To post-filter these segments, we trained a gradient-boosted decision-tree classifier using LightGBM (https://github.com/Microsoft/LightGBM) in R to classify nuclei based on the segment parameters, such as volume, the diameter of an equivalent circle, ratio of the number of voxels in a segment to the number of voxels in a bounding box, principal axis lengths, convex volume in voxels, solidity of the segment and surface area, to eliminate detritus or close proximity nuclei, with an accuracy of 97% on a test set.

### Centromere counting

Centromere counting was done independently for each nucleus by extracting the convex hull as defined by the segmentation. We used a modification of a previously described point-source-detection algorithm (Jaqaman et al., 2008) applied to the CENP-A signal. We have previously extensively used CENP-A tracking to study chromosome dynamics in mitosis, during which cells round up, resulting in well-resolved fluorescence spots (Armond et al., 2015a,b; Armond et al., 2016, 2019 preprint; Vladimirou et al., 2013). However, interphase cells are relatively flat and, even though the majority of CENP-A puncta represent well-resolved centromeres, occasionally overlapping centromere signals produce larger and brighter spots. We therefore developed an algorithm to resolve the superimposed centromeres to a count. The algorithm first detects candidate point sources as local maxima, which are then filtered by thresholding to keep those candidates that are brighter than a robust estimate of the mean background intensity of the image plus 5 standard deviations. Next, the size of the point spread function (PSF) of a single centromere was estimated by fitting 3D Gaussian variable-sized PSF models to the point sources in the image and taking a robust estimate of the mean size. The size estimate was then used to refit the point sources with 3D Gaussian fixed-size PSFs. The intensity amplitude was extracted from each of these PSF fits and the distribution across the nuclei was modelled with a Gaussian mixture model with one to four components. The best model was selected using Bayesian information criterion. We found that, in all cases, the Gaussian mixture component with the smallest mean consisted of the majority of the point sources; therefore, we used this as the intensity standard for counting centromeres. Thus, a candidate twice as intense indicates two centromeres rather than a more intense source. Given this fixed intensity standard, we computed the total centromere count from all the fitted PSFs. We found that 90% of the points were resolved as single centromeres, whereas 10% comprised two superimposed centromeres. To validate our approach, we applied our automated centromere-counting algorithm on untreated RPE1 cells, and found that the modal count of centromeres was 45 with a spread of counts around the modal number.

### G2 cell elimination

Using centromere detection, we were able to additionally eliminate cells that had completed DNA replication and could not incorporate any EdU (late S or G2 cells). These cells would be EdU negative and have many sister-chromatid pairs. To eliminate these cells, we used the detected positions of the centromeres to compute the distribution of Euclidean distances between them. We then excluded from the analysis nuclei that had more than 20 sister-chromatid centromere pairs, defined as centromeres closer than 0.8 μm, close to the rest length of sister-chromatid pairs (Armond et al., 2015b).

### Micronuclei counting

To count micronuclei, we designed and trained a new CNN to automatically quantify MN occurrence directly from images (Fig. 1D). The network architecture was as follows: 2048×2048×30 pixel 3D fields were maximally projected in the *z*-axis to two dimensions and resized to 1024×1024 pixels. The image was then divided into four quadrants and each quadrant passed through a pre-trained MobileNetV2 network (Sandler et al., 2018), and the output of the 1280 channels was extracted. The channels for each quadrant were summed together followed by 7×7 average pooling, before being passed through linear bottleneck layers with channels 1280, 256, 128 and 64 to finally produce a scalar output. We modified our custom nuclear segmentation-labelling tool to accept point clicks and we labelled 2160 fields, splitting into 80% train images and 20% for validation. We trained the model using PyTorch (https://pytorch.org/) with a batch size of four using the Adam optimizer (included with PyTorch) with an initial learning rate of 0.01, decaying per epoch by a factor of 0.95, and weight decay 0.0001. Subsequently, the MN count was divided by the number of nuclear segments that were retained through the LightGBM segment-classification model to obtain an average number of micronuclei per nuclear segment.

### Statistical testing of centromere and micronuclei counts

To assess statistically significant differences in centromere counts between conditions, we first fit a kernel density estimate of the histogram to remove undue influence from the noise arising from the automated counting procedure. We calculated the mode and FWHM of the density, as these measures are robust to outliers. Furthermore, noting that the mode would not be expected to shift without transfection efficiencies in excess of 50% as the mode represents the most frequent value, we also computed the percentage of cells with a count deviating from a near (±2) mode value, similarly to Bakhoum et al. (2009). We computed an estimate of statistical significance non-parametrically using permutation tests (10,000 samples) and estimated confidence intervals using bootstrap (1000 samples). The original code from this study is available at GitHub (https://github.com/evladimirou/CIN-quantification).

## Supplementary Material

10.1242/joces.260031_sup1 Supplementary information
