# Supplementary material for: Modest increase of KIF11 expression exposes fragilities in the mitotic spindle, causing chromosomal instability
Source: J Cell Sci. 2022 Aug 30;135(17):jcs260031. doi: 10.1242/jcs.260031 (PMC10500341; doi:10.1242/jcs.260031)
Supplement: Supplementary information [file joces-135-260031-s1.pdf]

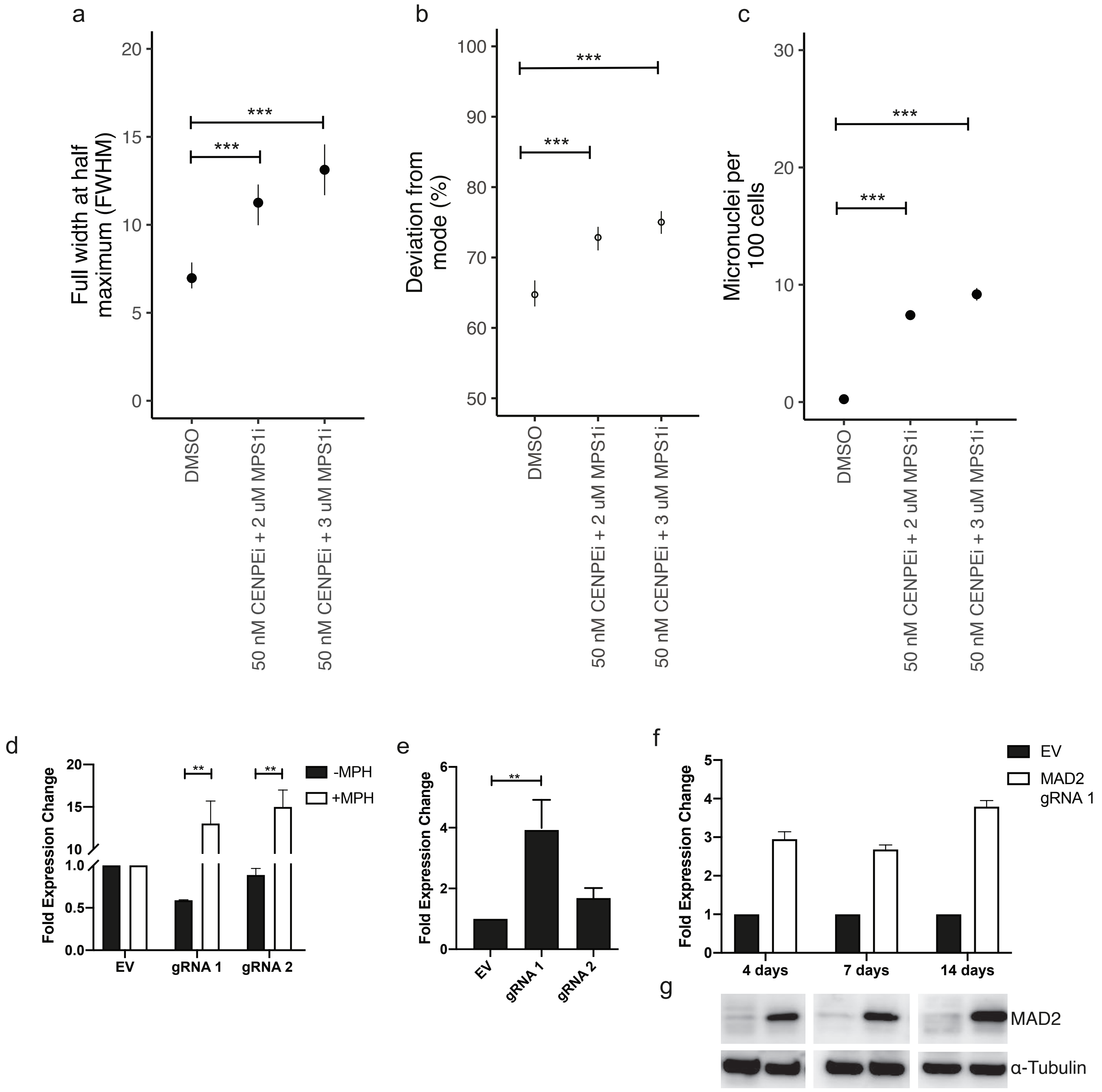

Fig. S1. Additional pharmacological validation of the CIN detection pipeline and comparison of gene upregulation schemes. (A) FWHM of centromere count frequency plots of DMSO and CENP-E and MPS-1 inhibitor treated RPE1-MPH cells (permutation test). (B) Percentage of centromere counts that deviate from the mode from DMSO and CENP-E and MPS-1 inhibitor treated RPE1-MPH cells (permutation test). (C) Number of micronuclei per 100 cells in DMSO and CENP-E and MPS-1 inhibitor treated RPE1-MPH cells (two-tailed, unpaired t-test). (D) Comparison of MAD2 upregulation in RPE1 cells, with (white) or without (black) co-transduction with pLenti-MPH (two-tailed, unpaired t-test). (E) Comparison of the efficacy of gRNAs targeting MAD2 in RPE1-MPH cells. (F,G) Timecourse showing sustained upregulation of MAD2 for up to 14 days post-transduction at the (F) mRNA and (G) protein levels. (*P<0.05, **P<0.005 , ***P<0.0005, error bars = SEM).

Journal of Cell Science • Supplementary information

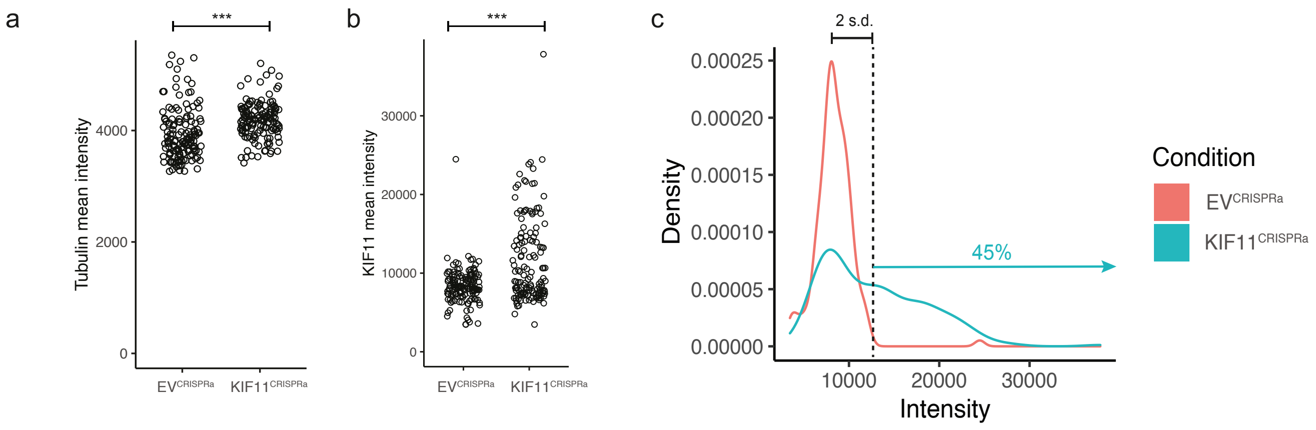

Fig. S2. Quantification of tubulin and KIF11 intensities in EV^{CRISPRa} and KIF11^{CRISPRa} and evaluation of KIF11 upregulation efficacy. (A,B) Tubulin mean intensities (A) and KIF11 mean intensities (B) from metaphase cells for EV^{CRISPRa} and KIF11^{CRISPRa} (two-tailed, unpaired *t*-test) n=145 EVCRISPRa, 140 KIF11^{CRISPRa}, 3 replicates. (C) Density plot of KIF11 signal intensities from EV^{CRISPRa} and KIF11CRISPRa cells. The vertical line shows the mean + 2 s.d. of the KIF11 intensity values of EV^{CRISPRa} to quantify the percentage of cell population with successful KIF11 upregulation in KIF11^{CRISPRa} cells at the protein level.

Journal of Cell Science • Supplementary information

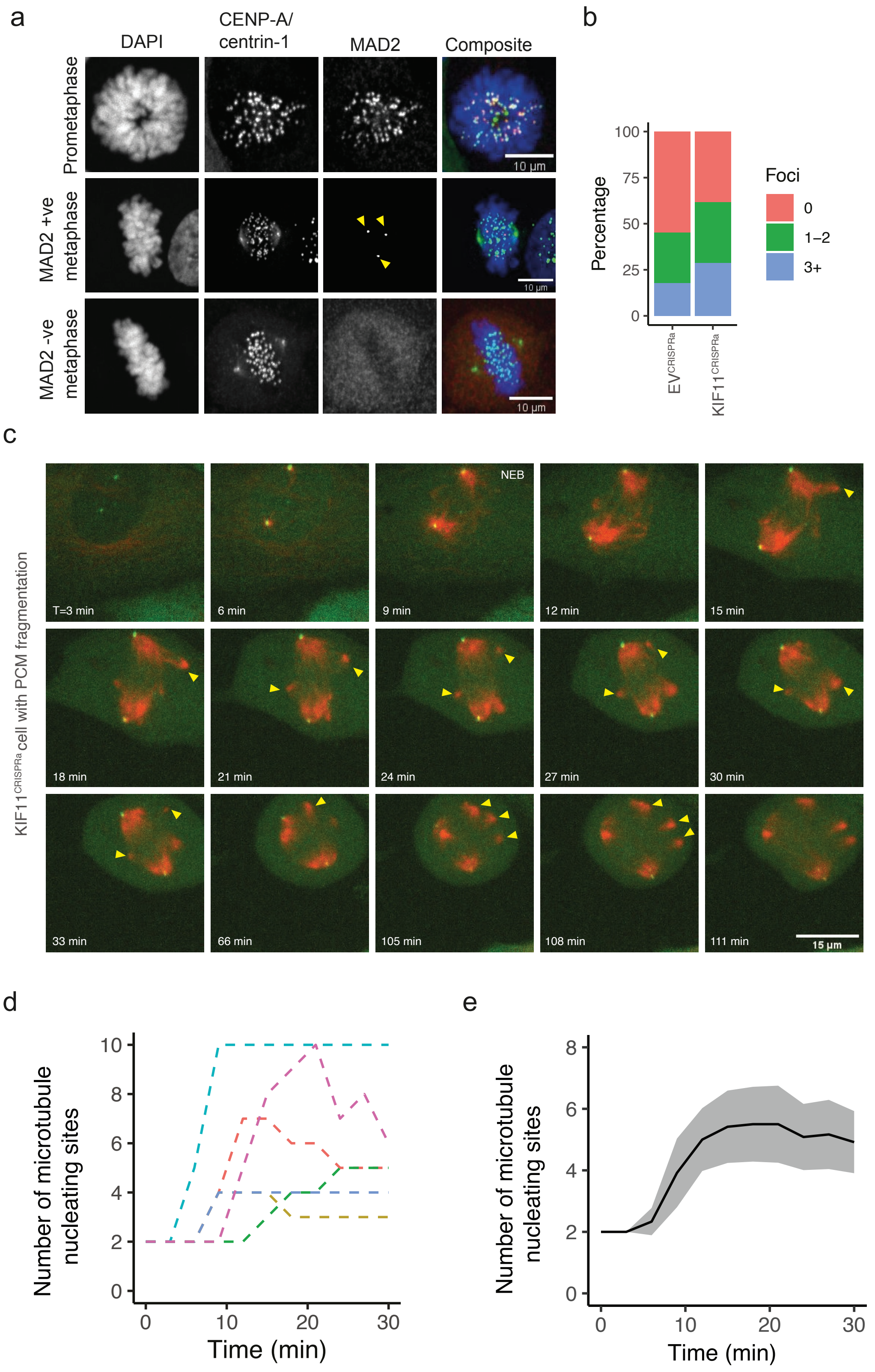

Fig. S3. Quantification of MAD2-positive kinetochores in $EV^{CRISPRa}$ and $KIF11^{CRISPRa}$ and timing of PCM fragmentation in $KIF11^{CRISPRa}$ cells. (A) Images of mitotic cells stained for MAD2 and CENP-A cells. Left-to-right: DAPI, CENP-A/centrin-1, MAD2, composite. Yellow arrow heads mark MAD2 foci. (B) Quantification of the number of MAD2 positive kinetochores in $EV^{CRISPRa}$ and $KIF11^{CRISPRa}$ cells (Fisher's exact-test). (C) Images from live imaging of a cell undergoing PCM fragmentation. SiR-tubulin shown in red, eGFP-centrin-1 shown in green. Yellow arrow heads mark extra SiR-tubulin foci. (D) Quantification of the number of microtubule nucleating sites in individual cells with respect to time, aligned at NEB. (E) Average number of microtubule nucleating sites from (D) with respect to time (grey band=sem).

Journal of Cell Science • Supplementary information

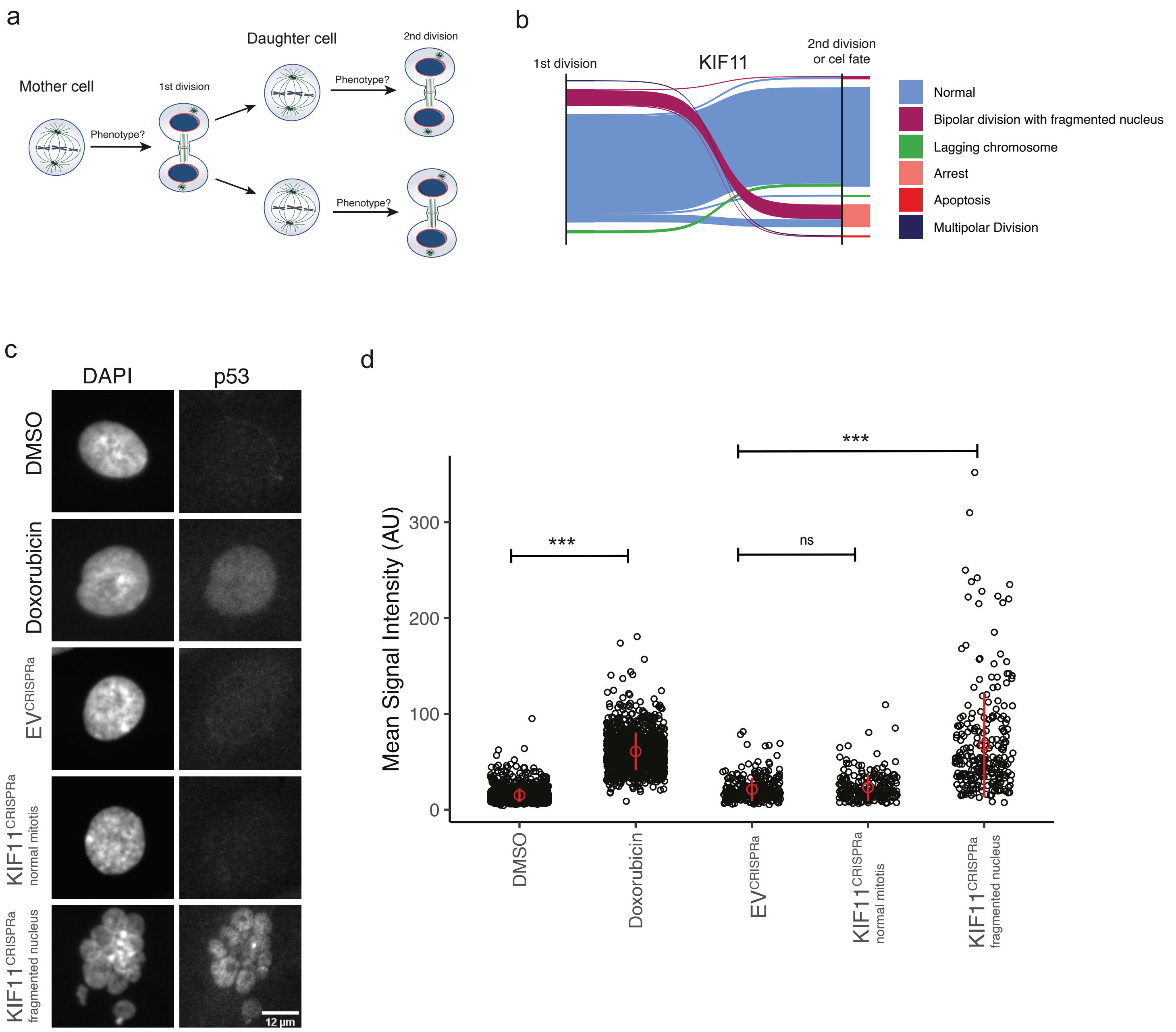

Fig. S4. Live cell imaging over multiple cell generations and cell fate classification. (A) Schematic for the phenotypic characterisation of the mitosis of two consecutive cell generations. Cells were imaged for a period of 60 hours using SiR-DNA to identify chromosomes and differential interference contrast to visualise the cytoplasm and cell cortex. (B) Cell fate classification as normal, bipolar division resulting in fragmented nuclei in interphase, multipolar division, lagging, arrest and apoptosis for two consecutive cell generations. The first time point shows the type of division for mother cells and the second time point shows either the cell fate of mother cell (if it didn't divide again) or the type of division for the daughter cells. (C) Example images of $EV^{CRISPRa}$ and $KIF11^{CRISPRa}$ cells stained for p53 along cells treated with doxorubicin (positive control) and DMSO (vehicle). (D) Quantification of the mean nuclear p53 signal intensity for DMSO, Doxorubicin, EV-transduced cells, $KIF11^{CRISPRa}$ cells with normal nuclei and $KIF11^{CRISPRa}$ cells with fragmented nuclei (two-tailed, unpaired t-test) (error bars = mean ± standard deviation).
